# Extracellular Vesicles Contribute to the Difference in Lipid Composition between Ovarian Follicles of Different Size Revealed by Mass Spectrometry Imaging

**DOI:** 10.3390/metabo13091001

**Published:** 2023-09-09

**Authors:** Emilie Maugrion, Ekaterina N. Shedova, Rustem Uzbekov, Ana-Paula Teixeira-Gomes, Valerie Labas, Daniel Tomas, Charles Banliat, Galina N. Singina, Svetlana Uzbekova

**Affiliations:** 1 CNRS, INRAE, University of Tours, PRC, 37380 Nouzilly, Franceana-paula.teixeira@inrae.fr (A.-P.T.-G.); valerie.labas@inrae.fr (V.L.); daniel.tomas@inrae.fr (D.T.); 2 PIXANIM, INRAE, University of Tours, CHU of Tours, 37380 Nouzilly, France; 3 L.K. Ernst Federal Research Center for Animal Husbandry, 142132 Podolsk, Russia; 4 Laboratory of Cell Biology and Electron Microscopy, Medical Faculty, University of Tours, 37032 Tours, France; 5 Faculty of Bioengineering and Bioinformatics, Moscow State University, 119992 Moscow, Russia; 6 Ecole Supérieure d’Agricultures (ESA), 49007 Angers, France

**Keywords:** MALDI-TOF mass spectrometry, molecular imaging, lipids, ovary, extracellular vesicles, follicular fluid

## Abstract

Follicular fluid (FF) ensures a safe environment for oocyte growth and maturation inside the ovarian follicle in mammals. In each cycle, the large dominant follicle (LF) contains the oocyte designated to be ovulated, whereas the small subordinate follicles (SFs) of the same wave will die through atresia. In cows, the oocytes from the SF, being 2 mm in size, are suitable for in vitro reproduction biotechnologies, and their competence in developing an embryo depends on the size of the follicles. FF contains proteins, metabolites, fatty acids, and a multitude of extracellular vesicles (ffEVs) of different origins, which may influence oocyte competence through bidirectional exchanges of specific molecular cargo between follicular cells and enclosed oocytes. FF composition evolves along with follicle growth, and the abundance of different lipids varies between the LF and SF. Here, significant differences in FF lipid content between the LFs and SFs within the same ovary were demonstrated by MALD-TOF mass spectrometry imaging on bovine ovarian sections. We then aimed to enlighten the lipid composition of FF, and MALDI-TOF lipid profiling was performed on cellular, vesicular, and liquid fractions of FF. Differential analyses on the abundance of detected lipid features revealed specific enrichment of phospholipids in different ffEV types, such as microvesicles (MVs) and exosomes (Exo), compared to depleted FF. MALDI-TOF lipid profiling on MVs and Exo from the LF and SF samples (*n* = 24) revealed that more than 40% of detected features were differentially abundant between the groups of MVs and Exo from the different follicles (*p* < 0.01, fold change > 2). Glycerophospholipid and sphingolipid features were more abundant in ffEVs from the SFs, whereas different lysophospholipids, including phosphatidylinositols, were more abundant in the LFs. As determined by functional analysis, the specific lipid composition of ffEVs suggested the involvement of vesicular lipids in cell signaling pathways and largely contributed to the differentiation of the dominant and subordinate follicles.

## 1. Introduction

Lipid metabolism plays a crucial role in reproductive functions by affecting female fertility in relation to nutrition, physiological state, and health, and at the ovarian level via the follicular environment of the female gamete, the oocyte. In mammals, an oocyte develops inside the follicle consisting of different types of somatic cells and follicular fluid (FF), which is produced from plasma and secretions by follicular cells. Different types of lipid bilayer-delimited nanoparticles, named extracellular vesicles (EVs), are released to FF, and these ffEVs participate in metabolic exchanges between the follicular cells and enclosed oocyte, which is crucial for the acquisition of oocyte competence to ovulate and develop viable embryos after fertilization [1,2,3]. According to the origin and size, EVs include extracellular microvesicles (MVs)—100–1000 nm particles formed by outward budding and fission of the plasma membrane, and 20–150 nm nanovesicles, also defined as exosome-like vesicles or exosomes (Exo) formed by the inward budding of the endosomal membrane to organize multivesicular bodies, which then fuse with the plasma membrane to release Exo [4]. EVs have diverse and dynamic lipid composition, which changes in response to various stimuli and consecutive metabolic stress. Within the ovarian follicles, Exo plays an important role during oocyte growth and maturation by carrying bioactive molecules of different types (miRNA, proteins, and lipids), which could also be the markers and response elements of external and internal stressors [5,6].

The lipid composition of EVs is thought to play a crucial role in their biological functions, including intercellular communication, immune modulation, and disease pathogenesis [7]. In cattle, in each cycle, a dominant follicle becomes the largest one and is designated to ovulate, whereas several subordinate follicles will go through atresia. In reproductive biotechnologies, bovine oocytes from the large follicles (LFs, diameter > 8 mm) showed a higher rate of in vitro embryo development after in vitro fertilization compared to the oocytes from the small follicles (SFs, 2–6 mm) [8,9]. Follicular growth is accompanied by proliferative, apoptotic, and steroidogenic functional activities of follicular cells [10] and by significant modulations of FF concentrations of different lipoproteins [11], glucose, fatty acids, and diverse metabolites [12,13]. In bovine ovarian follicles, FF showed the highest variation of lipid composition along with follicle growth: while less than 2% of lipids varied between the oocytes from LFs and SFs, 55% of FF total lipids changed their abundances in dominant LFs compared to subordinate SFs [14]. Follicle size and metabolic state of cows should also influence concentrations of ffEVs and their miRNA and protein cargo [15].

Follicular cells and FF shared about two-thirds of lipid features; however, the cells contained more glycerolipids, phosphatidylethanolamines (PE), phosphatidylserines (PS), and ceramides, whereas the fatty acyls (free FA and carnitine), cholesterol ester (CE), phosphatidylinositol (PI), and lysophospholipids, which were lysophosphatidylcholines (LPCs), LPI, and LPE, were over-represented in FF [16]. Spatial distribution of lipid species through the ovarian sections could be obtained by mass spectrometry imaging (MSI) that allows a kind of “molecular histology” through differential distribution and abundance of the detected ions in situ. MSI is a very powerful tool to access different biomolecules at the cellular level in their original environment by keeping their spatial arrangement within the tissue [17]. Matrix-assisted laser desorption/ionization time-of-flight mass spectrometry (MALDI-TOF MS) is routinely used for MSI of different biomolecules, including lipids. Depending on the matrix composition and ion acquisition mode (positive or negative), MS spectra could be acquired in different mass-to-charge ratio (*m*/*z*) ranges and consist of a variety of substances (*m*/*z* species or features) with different intensities [18]. The distribution of the molecules is visualized as two-dimensional ion density maps and depends on the spatial resolution and sensitivity of the MS instrument. Application of MSI in the 100–1000 *m*/*z* range to ovarian sections revealed a differential distribution of numerous lipids through the ovarian compartments and discriminated the follicles from stroma and/or luteal bodies in mice, pigs, and cows [16,19,20,21]. Moreover, MSI allowed fine discrimination of follicular cell layers from FF inside the follicles [16,20], which was confirmed by differential MALDI-TOF MS lipid profiling on follicular cells and FF collected from the individual follicles [14,16]. Nevertheless, the origin of FF lipid composition differences between the follicles within the same organ is not clear and needs to be enlightened.

The aim of the present study was (i) to explore the lipids in ovarian follicles of different sizes by using MSI, (ii) to investigate lipid composition in cellular, vesicular, and liquid fractions of FF, and (iii) to compare lipid profiles of ffEVs between the large dominant and small subordinate follicles in cows.

## 2. Materials and Methods

### 2.1. Ethics

No experiments on live animals were performed: ovaries of cows (*Bos taurus*) were obtained from a local commercial slaughterhouse and used for collection of biological material from reproductive tracts of slaughtered cows.

### 2.2. Chemicals

Unless indicated, all of the chemicals were purchased from Sigma-Aldrich (Saint-Quentin Fallavier, France). Internal standard mix for lipid identification was purchased from Avanti Polar Lipids, Inc (Alabaster, AL, USA). LC-MS grade water (H_2_O) and methanol (MeOH) were purchased from VWR International (Plainview, NY, USA).

### 2.3. Biological Materials

Bovine ovaries from adult Holstein cows were provided from the local slaughterhouse. Within 3 h after sampling, the ovaries were transported on ice to the laboratory. Several ovaries carrying both small and large follicles (SFs and LFs, respectively) were frozen under liquid nitrogen stream and kept at −80 °C to use for mass spectrometry imaging (MSI).

Follicular fluids (FFs) were accurately aspirated from the small follicles (SFs, 3–7 mm in diameter) and from the large follicles (LFs, >8 mm in diameter) of 24 freshly collected ovaries separately, using a 21-gauge needle attached to a 1 or 5 mL syringe, avoiding blood contamination. Collected FFs were allocated to independent tubes for each follicle size on ice. Follicular cells (Cells) were removed from collected FFs by centrifugation for 10 min at 300× *g* at 4 °C. Cell-free fluid (FF1) was centrifuged for 15 min at 2000× *g* and 4 °C to remove the apoptotic bodies (ABs). Supernatant fluid (FF2) was centrifuged for 30 min at 12,000× *g* to remove the microvesicles (MVs). To pellet small extracellular nanovesicles (exosome-like ffEVs, Exo), MV-free fluid (FF3) was centrifuged at 100,000× *g* for 90 min at 4 °C (Beckman model L8-M with SW-55-Ti rotor, adjusted k-factor: 163, using the formula k = (2.533 × 10^11^) × ln(rmax/rmin)/RPM^2^, were r(min) = 60.8 mm and r(max) = 108.5 mm at 30,000 RPM). Thus, ffEV-cleared fluid FF4 was obtained. MVs and Exo were washed with 20 mM Tris-HCl buffer, recentrifuged at 12,000× *g* and 100,000× *g*, respectively, and the pellets were resuspended in Tris-sucrose solution (260 mM sucrose 20 mM Tris-HCl pH 6.8), as earlier described [14]. Protein abundance in the samples was estimated by protein quantification of 10-fold dilutions of ffEV preparations and 100-fold dilutions of FF in water, using Bicinchoninic acid Assay (BCA; Interchim, Montluçon, France) according to manufacturer’s instructions using BSA as a standard.

### 2.4. Mass Spectrometry Imaging (MSI) by MALDI-TOF

Whole frozen bovine ovaries were put to a Cryo-Star HM 560 cryostat (Microm, Francheville, France) at −18 °C for 1 h, cut on 10 µm thick sections, and mounted onto conductive Indium Tin Oxide-coated slides (Bruker Daltonics, Wissembourg, France). All the sections were scanned using a histology slide scanner (OpticLab H850 scanner, Plustek, Ahrensburg, Germany). Selected slides were coated with the HABA (2-4-Hydroxyphenylazo benzoic acid) matrix sprayed at 1.5 mg/mL dissolved in 125 mM NH_4_2SO_4_ in 50/50 acetonitrile/H_2_O in the presence of 0.2% trifluoroacetic acid using an Image Prep device (Bruker Daltonik GmbH, Bremen, Germany). For external mass calibration, 1 μL of a mixture of small molecules and peptides (1 µL of calibratesolution containing Caffeine, MRFA peptide, Leu-Enkephalin, Bradykinin 2–9, Glu1-fibrinopeptide B; Reserpine; Bradykinine; Angiotensine I) was mixed (1:1 *v*/*v*) with the matrix employed for the MALDI MSI. After vacuum-drying for 24 h, spectra were acquired using an UltrafleXtreme MALDI-TOF instrument (Bruker Daltonik GmbH, Bremen, Germany) equipped with a Smartbeam laser (Nd:YAG, 355 nm) monitored by the FlexControl software version 3.4 (Bruker Daltonics, Bremen, Germany). MSI sequence was performed from whole ovarian section, targeting the regions with individual antral follicles. Spectra were acquired in the positive mode at a 2.0 kHz laser repetition rate in the 200–2000 *m*/*z* range, using a spatial resolution set at 40 µm (medium focus setting). A total of 250 spectra per pixel were collected as a sum of 25 consecutive laser shots in 10 random walk steps. The unprocessed MSI sequences were then imported into the SCiLS Lab software (version 2016b, SCiLS, GmbH, Bremen, Germany). MSI sequences were loaded and pre-processed; data were baseline reduced with the setting of 20 for peak width using a convolution algorithm and normalized to the total ion count. For 2D visualization of each ovary section, 2D ion density maps using minimal interval width ± 0.2 Da and medium denoising were created from the average projection spectrum. Bisecting k-means clustering (using the correlation distance metric) was used for data partitioning to provide segmented images showing the regions of spectral similarity.

### 2.5. Transmission Electron Microscopy (TEM) Analyses of Follicular Fluid Extracellular Vesicles

Transmission electron microscopy was used to characterize the preparations of ffEVs (MVs and Exo). TEM analysis was performed on six independent samples per class using the aliquots from each EV preparation fixed in 1% glutaraldehyde in PBS (pH 7.4).

The procedures were performed at room temperature unless otherwise specified. For each sample, 3 µL of fixed EVs was placed on Formvar/carbon-coated nickel grid (100 hexagonal mesh) and incubated for 1 h in humid chamber. For negative contrast staining, sample grids were washed with distilled water (three times for 10 s) and contrasted with a 2% water solution of uranyl acetate (three times for 10 s). The last drop of uranyl acetate was removed with filter paper, and the samples were air-dried.

For ultrastructural analysis of ffEVs, the pellets were fixed in a mixture of 4% formaldehyde and 1% glutaraldehyde in 0.1 M phosphate buffer (pH 7.4). Samples were washed 3 times for 30 min in 0.1 M phosphate buffer, then kept in the same conditions for 12 h to remove the traces of fixator, and then post-fixed with 2% osmium tetroxide (Electron Microscopy Science, Hatfield, PA, USA) in 0.15 M phosphate buffer for 1 h. Then, the samples were washed two times for 10 min in 0.1 M phosphate buffer, rinsed twice for 10 min in distilled H_2_O, and dehydrated through a gradual series of ethanol solutions (2 × 10 min in 50% ethanol in H_2_O and 3 × 15 min in 70% ethanol; 3 × 20 min in 90% ethanol; and 3 × 20 min in 100% ethanol). Final dehydration was performed in 100% propylene oxide (PrOx, TermoFisher GmbH, Kandel, Germany) for 3 × 20 min. Then, the samples were incubated in PrOx/EPON epoxy resin mixture (Sigma-Aldrich, St Louis, MO, USA) in a 2:1 ratio for 2 h, in PrOx/EPON epoxy resin mixture in a 1:2 ratio for 2 h, with closed caps, and 16 h with open caps, and finally in 100% EPON resin for 24 h. Samples were included in fresh 100% EPON resin and incubated at 37 °C for 24 h and then at 60 °C for 48 h for polymerization. Ultrathin 70 nm sections were cut using “Leica Ultracut UCT” ultramicrotome (Leica Microsystems GmbH, Wien, Austria) and stained for 20 min with 5% uranyl acetate (Electron Microscopy Science, Hatfield, PA, USA), then 5 min with lead citrate, and finally placed on TEM nickel one-slot grids (Electron Microscopy Science, Hatfield, PA, USA), coated with Formvar film. The micrographs were obtained using JEM 1011 (JEOL, Tokyo, Japan) equipped with a Gatan digital camera driven by Digital Micrograph software (Gatan, Pleasanton, CA, USA) at 100 kV. The images were used to measure nanoparticle size using ImageJ software (NIH, Bethesda, USA). Comparison of MVs and Exo size distribution was carried out using chi-squared test by XLSTAT (Addinsoft, Paris, France).

### 2.6. Lipid Profiling of Follicular Fluid Fractions by MALDI-TOF MS

To analyze FF-depleted fractions, non-diluted samples were taken. For analysis of non-fluid fractions, the pellets of cells and ffEV-enriched fractions were resuspended in Tris-sucrose solution (260 mM sucrose, 20 mM Tris-HCl, pH 6.8). Suspensions of the cells and extracellular elements were sonicated for 1 min using an ultrasonic bath (FisherBrand 15,052, Fisher Scientific). An amount of 1 microliter of the samples was overlayed by 1 microliter of 100% methanol and 1,5 microliters of freshly prepared DHAP (2,5 Dihydroxyacetophenone) matrix at 20 mg/µL dissolved in 90% methanol/9.8% water in presence of 0.2% TFA (trifluoroacetic acid) onto a MTP Polished 384 MALDI plate (Bruker Daltonics, Germany). Three technical replicates were spotted with the dried droplet technique. The matrix/sample mix was allowed to evaporate at room temperature for 15 min before MALDI-MS analysis. All spectra were acquired during the 30 min following the evaporation, using RapifleX MALDI Tissuetyper TOF mass spectrometer (Bruker Daltonics, Germany) equipped with a Smartbeam 3D Nd:YAG (355 mm) laser and FlexControl software (v4.0). M5 defocus smart beam parameter was set at a 30 µm × 30 µm scan range with a resulting field size of 109 µm. Spectra were obtained in positive and negative reflectron ion mode in the *m*/*z* 100–1200 range at 5 kHz laser repetition rate, with a sampling rate of 1.25 GS s^−1^. Ionization was achieved using a fixed laser power adjusted according to on-sample test shots in order to reach the optimal ionization threshold. Each spectrum was collected as a sum of 1500 laser shots in a random walk on spot. Each sample was analyzed in triplicate.

The parameters used for spectra acquisition were ion source 1, 19.96 kV; Electrode P2, 1.60 kV; lens, 8.956 kV; and a pulsed ion extraction of 200 ns. The “Detector check” function was conducted in order to obtain a detector gain at appropriate voltages. For the measurements, the instrument was externally calibrated in Quadratic mode using a mixture of peptides and proteins. For acquisition in positive mode, calibration solution contained DHAP [M + H]+ = 153.15 *m*/*z*, Phosphocholine [M + H]+ = 184.07 *m*/*z*, MRFA [M + H]+ = 524.26 *m*/*z*, PC:20.0 [M + H]+ = 566.38, PC:20.0 [M + Na]+ = 588.36, PC:20.0 [M + K]+ = 604.33, Bradykinin 2-9 [M + H]+ = 904.46 *m*/*z,* and Bradykinin [M + H]+ = 1060.56 *m*/*z*. For negative mode, the calibration solution contained DHAP [M − H]− = 151.15 *m*/*z*, MRFA [M − H]− = 522.26 *m*/*z*, PC:20.0 [M − H]− = 564.38, Bradykinin 2-9 [M − H]− = 902.46 *m*/*z,* and Bradykinin [M − H]− = 1058.56 *m*/*z*.

To increase mass accuracy (mass error < 0.05%), internal calibration using a lock mass at *m*/*z* 760.5851 (PC34:1) for positive ion mode and *m*/*z* 885.5499 for negative ion mode was subsequently applied to all spectra, with flexAnalysis 4.0 software (Bruker) and FlexAnalysis Batch Process (Compass 2.0).

All spectra were exported in mzxml format and treated using R software (version 3.6.1). Spectral processing and statistical analyses were performed using the MALDIquant and MALDIquantForeign packages (v1.19.3 and v0.12) of the R software. The profile spectra were treated for baseline subtraction (SNIP method), smoothed by the Savitzky–Golay algorithm, and realigned using prominent peaks and normalization on intensity using the total ion count method. Peaks were detected in automatic mode using a total average spectrum. The precision of the acquisitions was determined by calculating the coefficient of variation (CV) from the normalized peak intensity values of the three technical replicates for each sample. Mean CV of the peaks was lower than 50%. As MS data did not pass the Kolmogorov–Smirnov test of normality, all lipidomic data were submitted to the non-parametric Wilcoxon test to identify changes between two conditions and to the Kruskal–Wallis test to identify changes between several conditions. *m*/*z* were considered differentially abundant between the groups with a *p*-value < 0.05.

Hierarchical clustering (with Spearman correlations) and principal component analysis (PCA) were performed using the gplots (v3.0.3) and FactoMineR (v2.1) packages of the R software and using XLSTAT Biomed 2018.5 (Addinsoft, France).

### 2.7. Lipid Annotation

The differential peaks were annotated using theoretical masses ± 0.05% from the LIPID MAPS^®^ lipidomics gateway database considering [M + H]+ and salt adducts as [M + Na]+ or [M + K]+ in positive mode. For the negative mode, masses were annotated considering [M − H]− and chloride adducts as [M + Cl]−. The isotopes were characterized using enviPat package (v2.4) of the R software. Follow-up annotation of *m*/*z* features from the MS spectra was based on lipid identifications obtained by high-resolution LC/MS on bovine follicular cells and fluids that were described earlier [16]. For each detected ion, LIPID MAPS database was checked first, and then putative annotations were compared to the specific database of lipids identified from bovine FF and follicular cells [16]. If the annotations overlapped, the lipid features from the specific database with the smallest *m*/*z* differences between observed and theoretical *m*/*z* were retained. The lists of possible lipid annotations were reported in Supplementary data.

### 2.8. Pathway Analysis

Lists of detected *m*/*z* or annotated differential lipids were analyzed using MetaboAnalyst 5.0 (www.metaboanalyst.ca, accessed on 14 August 2023) in order to enlighten functional pathways and provide enrichment of detected metabolites. When *m*/*z* values were submitted to functional analysis, they were ranked by the *p*-values, and mass tolerance was 5 ppm. For pathways analysis, the list of annotated lipids was submitted. The enrichment factor of the pathway was calculated as a ratio between the number of significant pathway hits and the expected number of compound hits within the pathway.

## 3. Results

### 3.1. Mass Spectrometry Imaging (MSI) on Ovarian Sections Confirmed the Differences in Follicular Fluid Lipid Abundances between the Follicles in the Same Ovary

**Figure 1:**
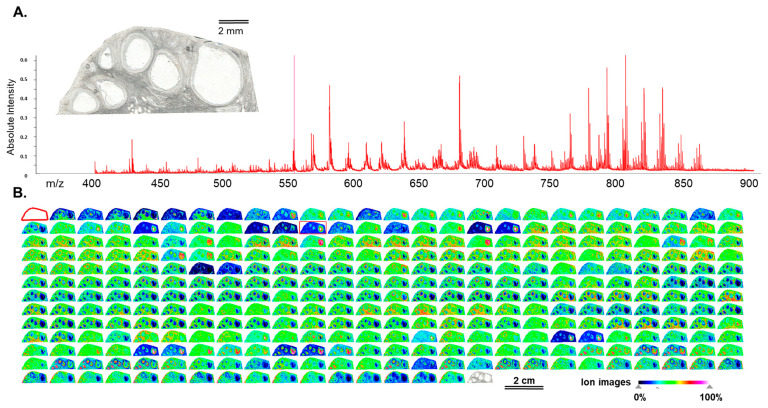
Matrix-assisted laser desorption/ionization time-of-flight (MALDI-TOF) mass spectrometry imaging (MSI) was used to detect lipid features on 10 µm sections of bovine ovary at a spatial resolution of 40 µm. (**A**) Light scan of frozen ovarian section and skyline projection spectrum of 327 molecular species in 400–900 *m*/*z* range. (**B**) Images of ion density maps of detected *m*/*z* species through ovarian section.

MSI was performed on frozen sections of bovine ovaries that contained one dominant follicle and several small ones (Figure 1). Overall, 327 peaks were detected in the 400–900 *m*/*z* range, as shown on the skyline projection spectrum (Figure 1A) and the images of ion density maps for each *m*/*z* (Figure 1B). A total of 203 *m*/*z* were annotated (Supplementary data, Table S1). Among the annotated lipids, glycerophospholipids PC and PE represented about 50% of all annotated features. About a quarter of all annotated *m*/*z* were the sphingolipids, mainly SM. More than 20% were lysophospholipids, represented by LPC and LPE. In addition, several carnitine ions were annotated (*m*/*z* 400.258 and *m*/*z* 428.274).

**Figure 2:**
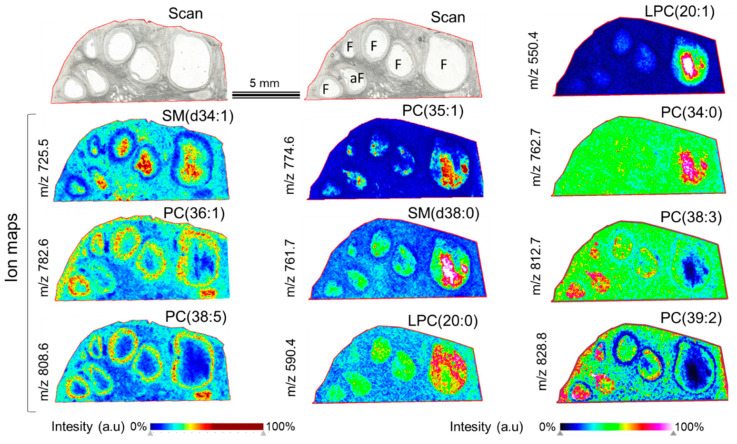
Detection of lipid species by MALDI-TOF MSI in bovine ovary. Images of light scan and ion density maps of several detected *m*/*z* species through the ovarian section. Tentative annotations of *m*/*z* are shown (see Supplementary data, Table S1). PC—phosphatidylcholine; PE—phosphatidylethanolamine; LPC—lysophosphatidylcholine; and SM—sphingomyelin.

Some representative images of ion density maps and their putative annotations are shown in Figure 2. Lipid ions were differently distributed through the ovarian section: some of them were present in both stroma and interstitial tissue and inside the follicles (*m*/*z* 590.4, LPC; *m*/*z* 725.5, SM). Other lipids were more intensively detected in FF compared to somatic tissues (like *m*/*z* 550.4 annotated as LPC; *m*/*z* 774.6—PC; and *m*/*z* 761.7—SM). Several PC species were mainly located in follicular cells (likely granulosa cell layers) and blood vessels (*m*/*z* 782.4 and *m*/*z* 808.5).

**Figure 3:**
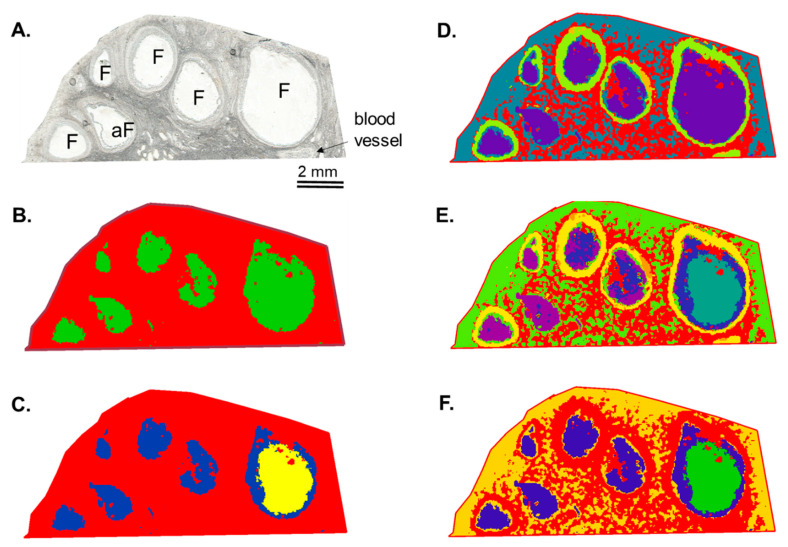
Lipidomic MALDI-TOF MSI from bovine ovarian section. (**A**) Light scan of analyzed ovarian section. (**B**–**F**) Segmentation maps obtained by hierarchical clustering of lipid profiles using the bisecting k-mean algorithms. Each image was obtained using different Euclidean distances and the number of spectra included in hierarchical clustering analysis. In each segmentation map, one color corresponds to similar lipid content. F—follicle and aF—atretic follicle.

Using hierarchical clustering, a variety of segmentation maps was created. The images of segmentation were generated using different numbers of spectra and different distances between the pairs of observations within two clusters (Figure 3). Thus, the image in Figure 3C was obtained from 9740 spectra, and the distance was 0.2600, whereas the image in Figure 3F was generated using 17,165 spectra, and the distance was 0.2104. Segmentation maps of different complexities evidenced that the differences in the lipid content were observed not only between the ovarian stroma and the follicles (Figure 3B) but also between different ovarian cell layers such as granulosa, theca, and stromal cells (Figure 3D,E). Moreover, the lipid profiles of FFs differed between the follicles (Figure 3C,F). Such clustering images were due to the differential spatial distribution of many lipid features more or less abundant between these ovarian compartments. In fact, ion maps of individual molecular species demonstrated that several lipids were over-abundant in follicular cells compared to FFs (as *m*/*z* 725.5, *m*/*z* 782.4, and *m*/*z* 808.5), whereas other substances prevailed in FFs (*m*/*z* 550.4, *m*/*z* 590.4, *m*/*z* 761.7, *m*/*z* 762.7, *m*/*z* 774.6, *m*/*z* 812.7, and *m*/*z* 828.8), as shown in Figure 2. Among the detected lipids, several ones were more abundant in the large dominant follicles compared to the smaller ones (as *m*/*z* 550.4, *m*/*z* 590.4, *m*/*z* 761.7, *m*/*z* 762.7, and *m*/*z* 774.6). In comparison, other lipids (*m*/*z* 812.7 and *m*/*z* 828.8) were more represented in the SFs compared to the large follicle, which was likely a dominant one.

### 3.2. Analysis of Lipids in Depleted Follicular Fluid Fractions

**Figure 4:**
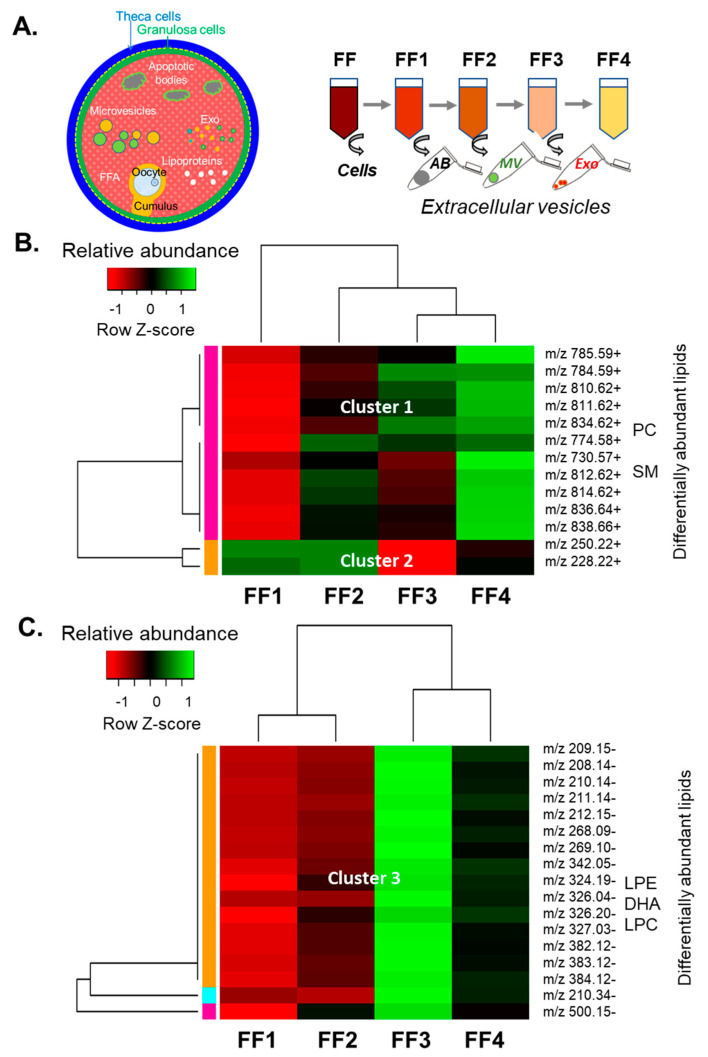
Differential analysis of lipid composition in depleted fractions of follicular fluid (FF). (**A**) Ovarian follicular fluid lipids are present in different forms, including albumin-coupled fatty acids (FFA), lipoproteins, apoptotic bodies, and extracellular vesicles. Workflow of FF depletion by removing cellular and extracellular components by applying differential centrifugation. ABs—apoptotic bodies; MVs—microvesicles; and Exo—exosome-like nanoparticles. FF1, FF2, FF3, and FF4—follicular fluid fractions consequently depleted from the cell ABs, MVs, and Exo, respectively. Relative abundance of differential lipids in FF fractions FF1-FF4, detected in positive and negative acquisition ion mode, presented as heatmaps (**B**,**C**), respectively. Lipid annotations are shown in Supplementary data, Table S2. Some lipid classes of identified *m*/*z* are shown on the side of heatmaps. DHA—docosahexaenoic acid; LPE—Lysophosphatidylethanolamine; LPC—lysophosphatidylcholine; PC—phosphatidylcholine; and SM—sphingomyelin.

In order to enlighten the differences in FF lipid composition between the follicles of different sizes observed by MSI, we characterized lipid fingerprints of different FF fractions obtained by sequential centrifugations by MALDI-TOF MS profiling (Figure 4A). Lipid profiling of the samples from FF1 to FF4 revealed 224 and 250 *m*/*z* peaks, detected in positive and negative acquisition ion modes, respectively, in the 200–1000 *m*/*z* range. Annotations were found for 134 *m*/*z* (+) and 41 *m*/*z* (−). Accordingly, the glycerophospholipids of PC and SM classes were the most abundant in FF fractions, whereas the PE, PI, and PS were the minor forms. Among the lysophospholipids, the LPCs were the most represented; however, LPE, LPI, and LPS were also detected (Supplementary data, Table S2).

Differential analysis of lipids between FF1, FF2, FF3, and FF4 preparations revealed 30 differentially abundant features (*p* < 0.05) (Supplementary data, Table S3). Abundancies of differential *m*/*z*(+) and *m*/*z*(−) peaks were presented as the heatmaps (Figure 4B,C, respectively), and three clusters were therefore determined. Lipids detected in positive ion mode (Cluster 1) progressively increased relative abundance from FF1 to vesicle-free FF4 and contained nine PCs and two SMs, according to lipid annotation. Two non-annotated features of Cluster 2 decreased their intensities along with centrifugation steps. Cluster 3 contained *m*/*z*(−) features, which were more abundant in the exosome-enriched FF3 fraction. Among them, two LPCs (*m*/*z* 326.04− and *m*/*z* 382.12−), LPE (*m*/*z* 500.15−) and docosahexaenoic acid C22:0 (*m*/*z* 327.03−) were annotated. It is worth noting that variations of relative abundances were relatively slight: the mean ratio FF4 to FF1 of all the features from the Cluster 1 was 1.79 ± 0.05 (Figure 4B), and the ratio FF3 to FF1 from the Cluster 2 was 0.39 for *m*/*z* 228.21(+) and 0.32 for *m*/*z* 250.21(+) (Figure 4B,C). Nevertheless, the mean ratio of FF3 to FF1 for 17 ions of Cluster 3 was 2.49 ± 0.16 (Figure 4C).

### 3.3. Analysis of Lipids in Cellular and Vesicle Fractions of Follicular Fluid

**Figure 5:**
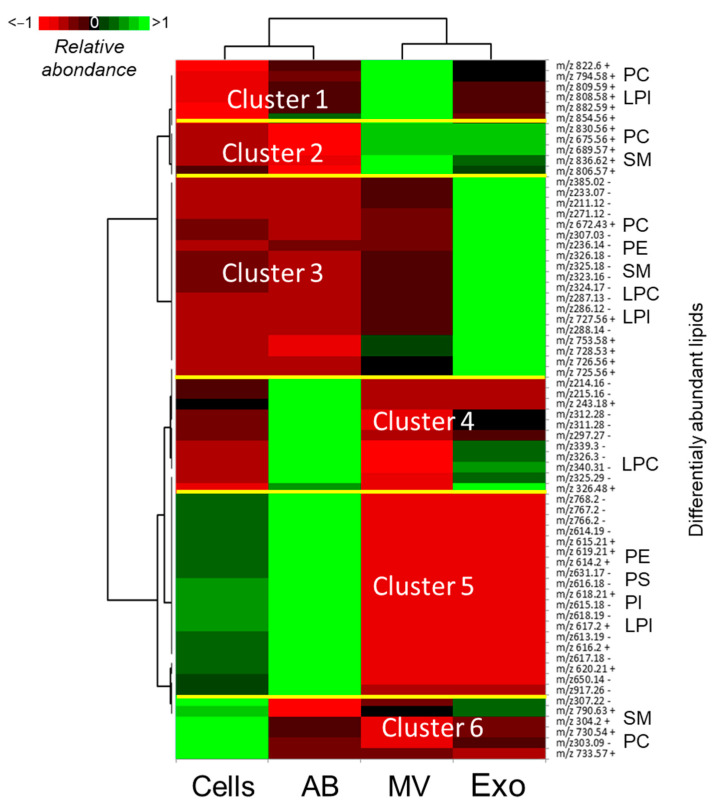
Differential analysis of lipids in different non-liquid fractions of bovine follicular fluid by MALDI-TOF MS profiling. Cells—free-swimming cells; ABs—apoptotic bodies; MVs—microvesicles; and Exo—exosome-like nanoparticles. Relative abundances of differential lipids in the Cells, ABs, MVs, and Exo presented as a heatmap. Lipid classes of annotated *m*/*z* within each cluster are shown to the right of the heatmap. Lipid classes of identified *m*/*z* are shown on the side of heatmaps. DHA—docosahexaenoic acid; LPE—Lysophosphatidylethanolamine; LPC—lysophosphatidylcholine; PC—phosphatidylcholine; and SM—sphingomyelin.

Non-liquid fractions, which were obtained after sequential centrifugations of FF, included free-swimming cells (Cells), cell remnants or apoptotic bodies (ABs), and ffEVs represented by MVs and Exo. Lipid profiles of different fractions of FF were analyzed using MALDI-TOF MS profiling. In total, 140 *m*/*z* and 186 *m*/*z* were detected in the Cells, ABs, and ffEVs in positive and negative ion acquisition modes, respectively, in the 100–1000 *m*/*z* range. In total, 202 *m*/*z* peaks were annotated (Supplementary data, Table S4). Differential analysis between the Cells, ABs, MVs, and Exo revealed 30 *m*/*z* (+) and 36 *m*/*z* (−) different intensities between these fractions (*p* < 0.05) (Supplementary data, Table S5). Relative abundances of these lipids were presented as a heatmap, and six clusters of lipid abundance variations were revealed (Figure 5).

Twenty-five differential lipids were annotated (Table 1). In fact, each *m*/*z* may also represent different lipid ions; different PC, SM, and LPI features were enriched in MVs and Exo compared to Cells and ABs (Clusters 1 and 2). Cluster 3 represented the lipids particularly overrepresented in Exo and included PC/PE, LPC, and SM. The lipids enriched in ABs were regrouped to Cluster 4 and included LPC (5:0). Cluster 5 represented the lipids, which demonstrated lower abundance in MVs and Exo compared to the Cells and ABs. This cluster included PE, PI, PS, and LPI (20:5). Cluster 6 included features overabundant in free-swimming cells precipitated from the FF compared to extracellular elements’ ABs, MVs, and Exo, and several *m*/*z* were annotated as PC and SM.

**Figure 6:**
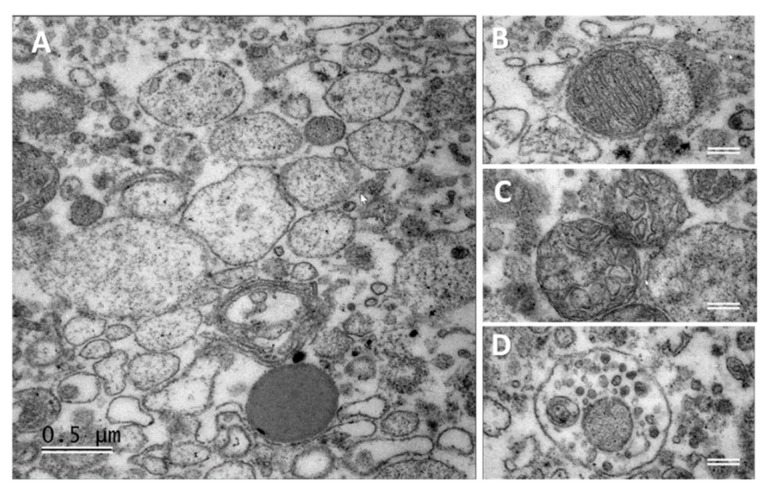
Fraction of microvesicles (MVs) extracted from bovine follicular fluid. Transmission electron microscopy was performed on ultrathin sections of MV pellet. Scale bars: (**A**)—500 nm and (**B**–**D**) 200 nm.

Since lipid profiles of ffEVs were significantly different from cellular fractions, morphological analysis by using transmission electron microscopy was performed on ultrathin sections of the fixed pellets after centrifugation of FF2 and FF3, which were enriched in MVs or Exo, respectively. Great variability in the morphology of MVs was observed (Figure 6). MV fraction contained different structures as free membrane-coated nanovesicles filled with filamentous structures (Figure 6A), debris of the organelles like mitochondria (Figure 6B) or endoplasmic reticulum (Figure 6C), or microvesicular bodies (Figure 6D).

**Figure 7:**
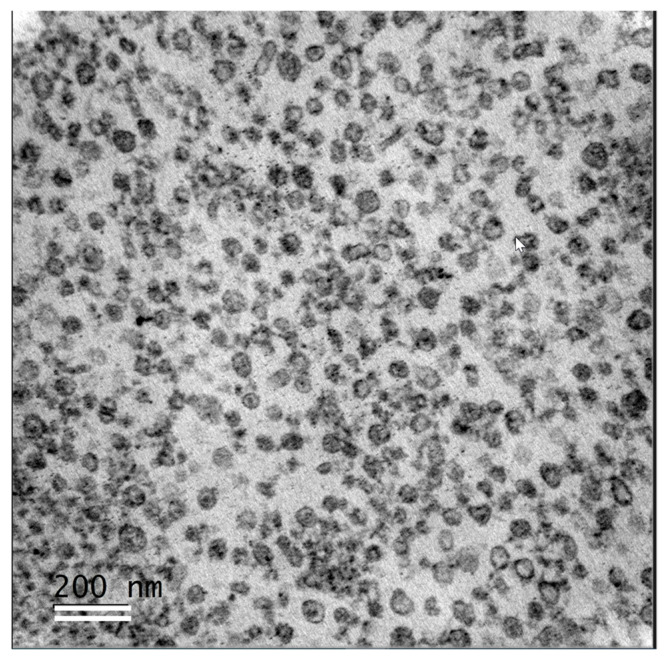
Fraction of exosome-like extracellular vesicles (Exo) extracted from bovine follicular fluid. Transmission electron microscopy was performed on ultrathin sections of Exo pellet. Scale bar: 200 nm.

Pellets after FF3 centrifugations were enriched in exosome-like EVs (Exo) and seemed to be more homogenous compared to MVs, although different forms and transparencies of nanovesicles were observed in Exo (Figure 7).

### 3.4. Analysis of Lipids in Follicular Fluid Extracellular Vesicles

**Figure 8:**
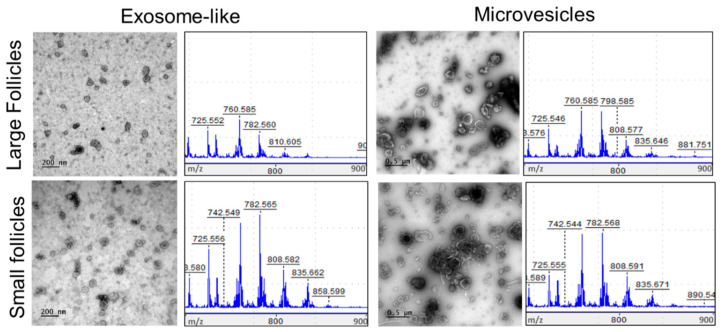
The fractions of FF exosome-like vesicles (Exo) and microvesicles (MVs) from the large follicles and small follicles were analyzed by MALDI-TOF MS. Transmission electron microscopy images of Exo and MVs are shown. Scale bars are 200 nm (Exosome-like images) and 500 nm (Microvesicles images). Representative images of row MALDI-TOF MS spectra in *m*/*z* range of lipids (*m*/*z* 700 – *m*/*z* 900) are shown.

Taking into account the differences in lipid composition between the SF and LF revealed by MSI, the fundamental role of ffEVs, and, notably, the exosomes in intercellular molecular exchanges and functional activities of follicular cells, we performed comparative analyses of lipids in the fractions enriched in both the ffEV types (MVs and Exo), extracted from the follicles of different sizes. Exosome-like vesicles (*n* = 600) prepared from follicular fluids of LFs and SFs had similar sizes (61.2 ± 0.08 nm in LFs and 58.6 ± 0.07 nm in SFs, respectively, *p* = 0.16), whereas MVs (*n* = 600) had larger diameters in the large follicles (LFs) compared to small follicles (SFs) (183.1 ± 6.0 nm, and 153.5 ± 4.2 nm, respectively, *p* < 0.001), according to the TEM analysis. These Exo and MV preparations were analyzed by MALDI-TOF MS, and their lipid fingerprints were obtained (Figure 8).

In total, from all the aligned spectra of MVs and Exo, 316 *m*/*z* were detected in positive (+) and 359 *m*/*z* in negative (−) acquisition ion modes. A total of 315 peaks from both modes were annotated (Supplementary data, Table S6). Although the annotation of several glycerophospholipids was ambiguous, the PC largely prevailed over PI, PE, and PS and together represented about 55% of total ffEV annotated lipids. Accordingly, the rate of their lysoforms was estimated at about 19% from all the annotated lipids, and LPC was the major form. SM represented approximatively 19% of lipids, CE, and ceramides represented 3.5% and 2.5% of lipids, respectively, and less than 1% were other lipids, including carnitine. The list of *m*/*z* peaks detected in ffEVs in positive and negative modes was analyzed through the MetaboAnalyst platform to attain functional pathways in which the detected features were involved. The results are reported in Table 2.

As indicated, the metabolites detected in MVs and Exo were linked to the different pathways related to energy metabolism, FA transformation and oxidation, and lipid signaling mediators.

### 3.5. Comparative Analysis of Lipids between MVs and Exo

**Figure 9:**
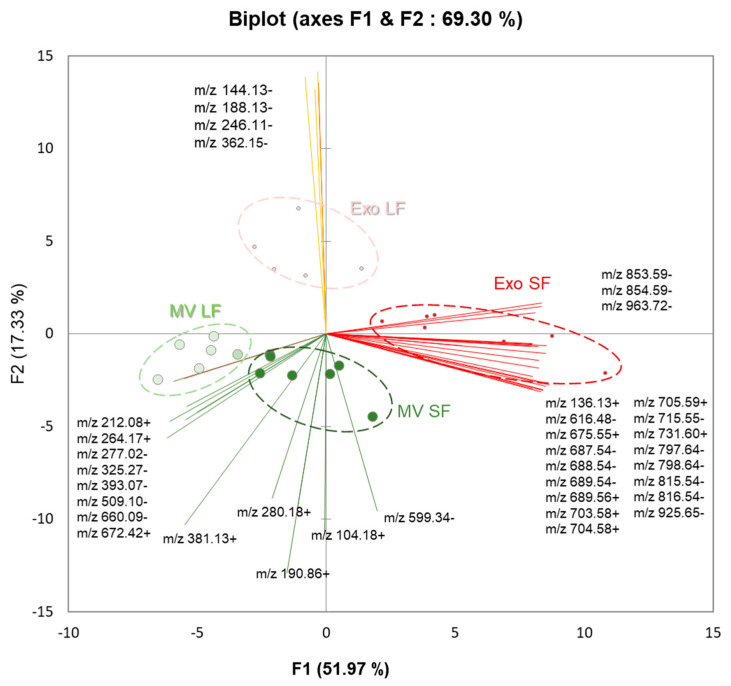
Differential analysis of lipids in bovine follicular fluid fractions of extracellular microvesicles (MVs) and exosome-like nanovesicles (Exo) from small follicles (SFs) and large follicles (LFs) by MALDI-TOF MS lipid profiling. Discrimination of MVs and Exo by their lipid profiles, by principle component analysis. Biplot showed the *m*/*z* features discriminating different groups of ffEVs.

When comparing MVs and Exo, regardless of the size of the follicles they were extracted from, only 37 ions were differentially abundant (*p* < 0.05, fold change > 2). Principle component analysis (PCA) using normalized intensity values of differentially abundant *m*/*z* species clearly discriminated MVs from Exo (Figure 9). Moreover, PCA also discriminated ffEVs by their follicle size, whether LFs or SFs.

Among the differential *m*/*z*, 24 features were over-represented in Exo, whereas 13 *m*/*z* were higher in MVs (Table 3). Different SM and PC, PI, and LPC were over-represented in Exo, whereas LPI, LPS, and choline were overabundant in MV preparations, regardless of their origin from large or small follicles.

### 3.6. Comparative Analysis of ffEV Lipid Abundance between the Large and Small Follicles

**Figure 10:**
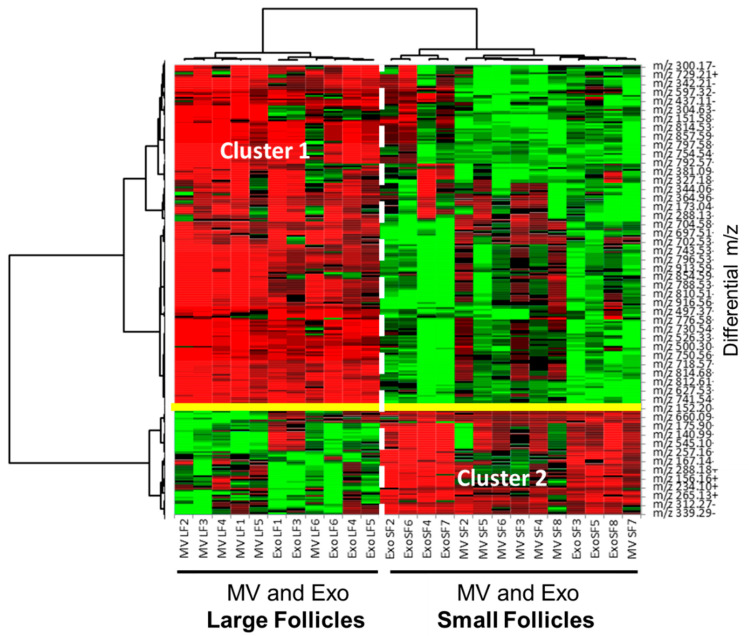
Differential analysis of lipids in bovine ffEVs by MALDI-TOF MS lipid profiling. Heat map representation of differentially abundant lipids (*p* < 0.05) detected in positive and negative acquisition modes in microvesicles (MVs) and exosome-like nanovesicles (Exo) extracted from either the large dominant follicles or small subordinate follicles.

MALDI-TOF lipid profiles of ffEVs from the follicles of different sizes (SFs and LFs) were then compared, regardless of EV type, by taking into account both MV and Exo samples from each follicle size group. Indeed, among the 512 significantly different *m*/*z* (*p* < 0.05), 36 *m*/*z* were more than two-fold over-abundant in the ffEVs from the LFs, and 268 *m*/*z* showed higher intensity in samples from the SFs (*p* < 0.05, FC > 2). Non-hierarchical clustering was performed on relative abundance values of differential lipids and was presented here as a heatmap (Figure 10). As shown, MV and Exo samples were grouped according to the size of the follicles from which they were isolated, although, inside the LF and SF groups, the Exo and MV samples were mixed. According to the heat map, two distinct clusters were evidenced: Cluster 1 regrouped the features that were significantly over-abundant in ffEV preparations from the SFs, whereas the lipids over-represented in the samples from the LFs were grouped in Cluster 2.

All differential lipids and their annotations are shown in Supplementary data, Table S7. The list of the most differential lipids is shown in Table 4.

According to lipid annotations, lysophospholipids LPC, LPS, and LPE were significantly more abundant in ffEV preparations from the LFs compared to SFs, whereas in the SFs, the lipids represented by SM, PE, PC, PI, P, LPE, LPI, and CE were more abundant.

The list of annotated differential lipids was analyzed using the MetaboAnalyst platform in order to determine functional pathways. By the results from this analysis, several pathways were enriched with the lipids differentially abundant between the LFs and SFs (Table 5). The most significant enrichment was shown for the lipids involved in Linoleic acid metabolism, Alpha-linolenic acid metabolism, and Glycosylphosphatidylinositol (GPI)-anchor biosynthesis. Metabolism of glycerophospholipids, sphingolipids, and phosphatidylinositol signaling system pathways demonstrated the most important impact values calculated through pathway topology analysis. In addition, pathways of phosphatidylinositol signaling and inositol phosphate metabolism were enriched in the ffEV lipids. Among the non-enriched pathways, the analysis revealed arachidonic acid metabolism and steroid biosynthesis pathways.

### 3.7. Comparative Analysis and Annotation of Lipids in MVs and Exo from the Large and Small Follicles

**Figure 11:**
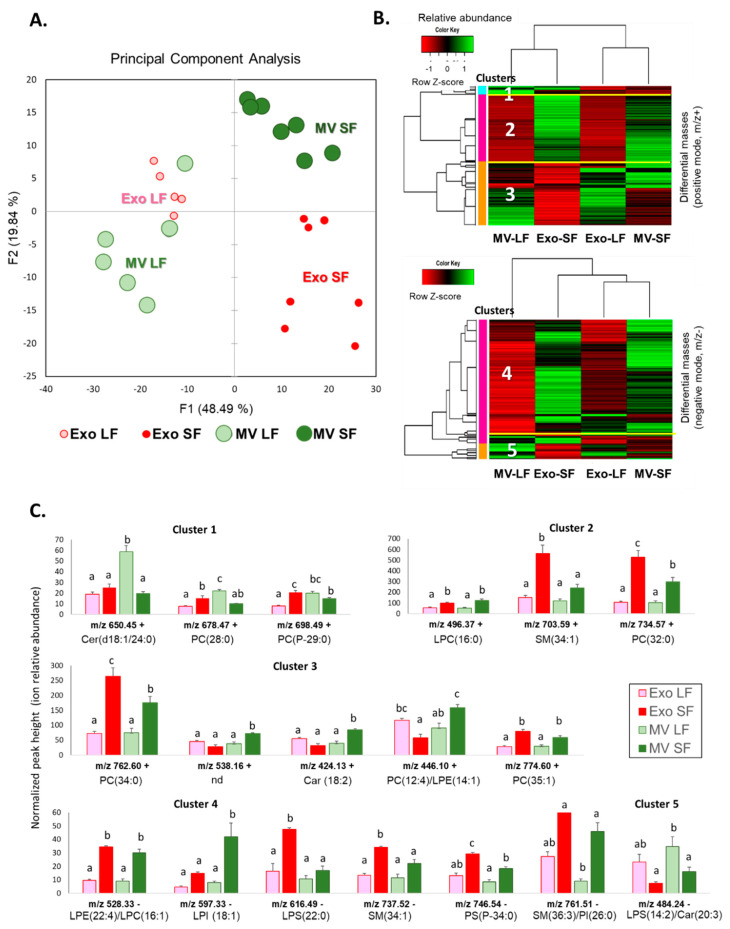
Differential analysis of lipid features’ intensities in extracellular microvesicles (MVs) and exosome-like nanovesicles (Exo) preparations from the large follicles (LFs) and small follicles (SFs) by MALDI-TOF MS lipid profiling. (**A**) Discrimination of MV LFs, MV SFs, Exo LFs, and Exo SFs by their lipid profiles, by principal component analysis. (**B**) Heatmap representation of differentially abundant lipids: *m*/*z* (+) (upper picture) and negative *m*/*z* (−) (lower picture) in MV LFs, MV SFs, Exo LFs, and Exo SFs (*p* < 0.01). (**C**) Relative abundance of several differential lipid species in MV and Exo preparations from the LFs and SFs, presented as mean normalized height +/− SEM. Different letters indicate significant differences (*p* < 0.05). LPE—Lysophosphatidylethanolamine; LPC—lysophosphatidylcholine; LPI—Lysophosphatidylinositol; PC—phosphatidylcholine; PE—phosphatidylethanolamine; PI—phosphatidylinositol; and SM—sphingomyelin.

Differential analysis using all lipid features detected in the MVs and Exo revealed 279 *m*/*z* (+) and 232 *m*/*z* (−) that were differentially abundant (*p* < 0.01) between the four groups of ffEVs originated from either small or large follicles: Exo SFs, MV SFs, Exo LFs, and MV LFs (Supplementary data, Table S8). Principle component analysis of normalized intensity values of differential features demonstrated clear discrimination of the follicles by their size (LFs or SFs). MV and Exo were also discriminated, especially in the SFs, whereas in the LFs, the lipid patterns of MVs and Exo were more similar (Figure 11A). Abundances of differential lipids in the groups were presented in two heatmaps (Figure 11B), and five clusters were determined. Clusters 1 to 3 contained *m*/*z* (+) features (Figure 11B, top picture), and Clusters 4 and 5 consisted of *m*/*z* (−) differential species (Figure 11B, bottom picture). A total of 232 differential *m*/*z* were annotated (Supplementary data, Table S9). Cluster 1 included Cer (d18:1/24:0), PC (28:0), and PC (P-29:0). Cluster 2 regrouped over-abundant lipids in both Exo and MVs from the SFs compared to the LFs. Among 100 annotated features of that cluster, there were mostly PC, SM, lysophospholipids (mainly LPC), and CE. Cluster 3 regrouped the lipids that were particularly overabundant in the Exo LF group and included two carnitines, two ceramides, and several PCs and LPCs (Supplementary data, Table S6). Cluster 4 represented the overabundant features in MV SFs and Exo SFs, similar to Cluster 2. Among the 114 *m*/*z* annotated, there were many PC, PI, and PE; the sphingolipids were represented by SM and Su. Fourteen differential lysophospholipids were LPC, LPE, LPI, and LPS. In addition, carnitine was also identified in Cluster 4. Cluster 5 regrouped the lipids, which were more abundant in the ffEVs from the dominant LFs and included LPI features.

The examples of abundance variations of identified lipids in MV and Exo fractions of FF from the follicles of different sizes are shown in Figure 11C. Globally, the preparations enriched in MVs and Exo from the LF demonstrated a relatively lower abundance of many lipids compared to the SF, except for the lipids from Clusters 3 and 5. Abundances of differential lipids in MVs and Exo varied between the SF and LF either in the same direction (as the lipids from Clusters 2 and 4) or in the opposite direction, where Exo and MV groups demonstrated different variation patterns between the LF and SF. The examples of difference between MVs and Exo were the lipids from Cluster 1 and several *m*/*z* from Cluster 3 (*m*/*z* 538.16, *m*/*z* 424.13, and *m*/*z* 446.10).

## 4. Discussion

In the presented study using MSI on bovine ovarian sections, it was demonstrated that large dominant follicles have significantly different lipid compositions compared to several subordinate follicles of the same ovary. This difference was mostly due to FF lipids present in cellular, vesicular, and liquid fractions of FF, which were analyzed by MALDI-TOF MS lipid profiling for the first time.

### 4.1. Mass Spectrometry Imaging of Bovine Ovary Revealed the Differences of Lipid Composition between the Large and Small Follicles

In the present study, we used MSI in positive ion mode to characterize lipid complexity within the ovarian follicles of adult cows. It was demonstrated that follicle-free interstitial stroma, follicular cell layers, and fluids were discriminated by their lipid composition in accordance with the previous MSI experiments reported in mice, pigs, and bovines [19,20,21]. In all these studies, different matrixes were used for MSI. Indeed, the matrix sprayed on tissue sections influenced the ionization efficiency of the molecules and, therefore, changed sensitivity for distinct subclasses of detected lipids [18]. Here, using a HABA-based matrix, 327 ion density maps were generated for bovine ovarian sections. In the porcine ovary, 2,5-dihydroxybenzoic acid (DHB) and α-cyano-4-hydroxycinnamic (CHCA) acid matrixes were used and allowed detection and quantification of 79 and 92 lipid ions through the ovarian sections, respectively [20]. In the bovine ovary, MSI using a CHCA-based matrix allowed the generation of high-resolution ion density maps for 281 ions from the spectra acquired in positive ion mode [16].

Hierarchical clustering of the MSI spectra generated different segmentation maps and thus formed molecular histology maps. Regardless of the matrix used, the segmentation maps demonstrated similar ovarian structures in all the species, consisting of the follicles and follicle-free stromal tissues, which had different lipid distributions [19,20,21]. Moreover, using a spatial resolution set at 22 µm that is compatible with an oocyte size, the enclosed oocyte could be distinguished due to its different lipid signature compared to follicular cells and fluids [20]. In addition, within the follicles, follicular mural theca and granulosa cell layers were also discriminated by MSI, similar to previous studies in porcine and bovine ovaries [16,20], reflecting different lipid composition and physico-chemical properties of these cell layers.

In the present study, using a spatial resolution set at 40 µm, we obtained different segmentation maps that discriminated not only the antral follicles from ovarian stroma but also the large dominant follicles from the smaller follicles. These differences were likely due to the very distinct lipid composition of the FF of SFs and LFs compared to lipid profiles of follicular cell layers, which seemed more homogenous between the follicles. However, refined analysis of lipid fingerprints of follicular theca and granulosa cells by MALDI-TOF MS showed 5.3% and 14.9% differences between the follicles of different sizes, respectively [14]. In contrast, 55% of FF lipids had significantly different abundance between LFs and SFs. In particular, several species of LPC, PC, CE, and SM were more abundant in FF from the dominant follicles compared to smaller ones, in contrast to numerous species of PE, PS, PI, and DG that were more abundant in the FF of SFs [14]. Indeed, FF is the most variable follicular compartment regarding its lipid profile, because FF reflects the metabolic composition of blood serum, which may change daily in relation to the periods of nutrition and fasting [22]. This study showed that FF varied between the follicles within the same ovary, which reflects the crucial importance of follicular lipid metabolism inside the follicle.

The lipid composition of FF in cows largely depends on the functional activities of the follicular granulosa and theca cells, which, in response to follicle stimulation hormone and other factors, change their steroidogenic, proliferative, and apoptotic activities along with follicle growth [23,24]. Significant variations in FF concentrations of free fatty acids [13], different lipoproteins [11,25], cholesterol, triglycerides, and glucose [12] were observed between the follicles of different sizes and steroidogenic activity. In our MSI analyses, several lipids, including LPC, PC, and SM, showed more intensity in the largest follicle compared to the smaller ones, as shown in the ion maps. LPC represented more than 3.5% of total lipids in the ovarian follicle [14,26], and in women, LPC concentration in FF changed in response to ovarian stimulation protocols and was associated with pathologies [27]. LPC are the most abundant lysophospholipids, integrated into different lipoproteins and known to mediate activation of different pathways, including mitogen-activated protein kinase ERKs, which are crucial in the regulation of follicle growth and oocyte maturation [28,29]. High LPC levels provided destroying effects on the cells by enhancing inflammatory response, disrupting mitochondrial integrity, and inducing apoptosis. Therefore, high LPC abundance in the LF might also be associated with a higher apoptosis rate in follicular cells and, consequently, more apoptotic bodies in the FF of the large follicles, compared to SFs, that reflected the lipid composition revealed by MSI.

Therefore, MSI of lipids in bovine ovarian sections could discriminate between the follicles of different sizes due to significant differences in the lipid composition of the internal antral space filled with FF, which contains different elements, including cells, extracellular particles, and also lipoproteins and fatty acids (FA) in different forms. To reveal the lipids responsible for these differences, FF fractions were then analyzed.

### 4.2. Lipid Distribution in Different Fractions of Follicular Fluid

FF contains free FA coupled with albumin and FA in esterified forms, integrated into the cells, apoptotic cell remnants, extracellular vesicles of different sizes and biogenesis, and lipoproteins. Here, we characterized different FF fractions by using MALDI-TOF profiling, which is a valuable approach in comparing numerous spectra obtained from very little biological material that does not have a long extraction procedure [30,31]. MALDI-TOF molecular profiling on intact cells, biological fluids, and crude tissue samples is widely used in reproduction research and veterinary medicine [32,33,34]. Although the MALDI-TOF profiling approach is not exhaustive in terms of the detection of lipid species, it allowed for comparison between many complex samples from individual follicles and even single oocytes and could reveal the lipids as candidate biomarkers related to oocyte competence [14,35,36,37,38,39,40,41].

According to our data, the most representative lipid classes in cell-free FF were PC, SM, LPC, LPE, and PE, in corroboration with the previous lipidomic data on follicular cells and fluid [16,26]. Several of these lipids are usually associated with biological membranes and could be a part of “solid” FF fractions like apoptotic bodies and ffEVs (MVs and Exo), as detected in the EVs released by different human cells [7]. We found that extracellular MVs and Exo showed significantly higher proportions of LPC and PI and a lower rate of SM compared to depleted FF. Although differences in lipid composition between MVs and Exo were evidenced, the most representative classes in both types of ffEVs were the PC, SM, LPC, PE, and PI, most of them being membrane building blocks. Cholesterol ester and ceramides were also detected. In different studies, it was reported that cholesterol accumulates in the EVs and impacts their fate and uptake by target cells [42]. Although present at a low rate, ceramides are also critical for EV formation and secretion [43].

In the present work, along with sequential centrifugations of FF and following depletions from the apoptotic bodies and ffEVs, only 6.3% of lipids changed their abundances since FF1 (cell-free FF with cell remnants and ffEVs) to FF4 fraction (FF depleted from ABs and ffEVs). These differential lipids included long-chain SM and PC, as well as short-chain LPE and LPC, and were enriched in FF fraction depleted from the extracellular particles (ABs and ffEVs). However, globally, FF before and after depletion showed relatively similar lipid patterns in terms of lipid subclasses. Probably, most of the FF lipids were associated with the elements, which were not precipitated by the centrifugations applied here. These were likely soluble lipoproteins, which generally contain a lipid core of triacylglycerol (TAG) and cholesterol ester (CE) and are surrounded by a phospholipid layer with embedded apolipoproteins in different proportions. In FF, the lipoproteins are represented mainly by the smallest 10–20 nm high-density lipoproteins (HDLs), which contain about 40% of proteins [44]. HDL concentration increased in the FF of the large follicles before ovulation [11]. Indeed, HDLs serve as a main reserve of cholesterol for steroidogenesis in follicular cells [44], and HDL cholesterol concentration in FF was correlated with oocyte quality and thus essential for female fertility [45]. Low-density lipoproteins (LDLs) and very low-density lipoproteins (VLDLs) are also present in very small proportions in FF [46] and, together with HDLs, play important roles in ovarian cholesterol transport [47].

The efficiency of detecting lipid classes depends on the matrix used for MALDI-TOF profiling. Here, using the DHAP matrix, several lipid classes were difficult to detect due to difficult ionization in the present conditions. Thus, among the annotated lipids in ffEVs, there were very few glycerolipids, whereas large amounts of TAG and DAG were identified in the EVs from blood serum, and the glycerolipid species were highly enriched in the EVs secreted by hepatocytes in humans [7]. The recent study by Da Silveira et al. reported the lipidome of bovine EVs from FF using mass accuracy hybrid quadrupole/TOF mass spectrometer Triple-TOF on extracted lipids [48]. Among 542 detected lipids, the PC and SM were also the most representative in MVs and exosomes at about 25% and 17%, respectively [48], similar to our findings. However, in contrast to our study, a high percentage of cardiolipins and TAG but not lysophospholipids were detected [48]. These discrepancies were certainly due to the preparation method, the matrixes and MS instruments used for ion detection, and the lipid annotation algorithm. Nevertheless, in agreement with different studies, which compared lipid composition between the MVs and exosomes in different cell sources [48,49,50], we also found significant differences in the relative abundance of PC, PE, LPC, LPI, and sphingolipids, including SM and ceramides. Although the mechanism of biogenesis of MVs and exosomes are coordinated, the pathways involved in the formation of these vesicles are different and result in their different membrane lipid composition, which affects membrane rigidity; thus, exosomes are more stable than MVs [51]. Analysis of lipids in MVs and exosomes from human mesenchymal and cancer cells revealed the specific lipid composition of these EV types, but globally, the exosomes were enriched in glycolipids and free FA, whereas MVs contained more SM and ceramides [49]. Indeed, the molecular composition of EVs largely depends on the cells that released them, but generally, EVs showed a two- to three-fold enrichment for sphingolipids, glycosphingolipids, cholesterol, and PS compared to the cells [52], whereas PC and PI were less abundant in EVs than in the cells [7]. In our study, PC(32:2), PI(40:2), LPI(20:5), and different PE were less abundant in the ffEVs compared to the cells and their remnants (ABs). In contrast, SM and PC were enriched in the MVs and Exo.

Taken together, comparing lipid profiles between FF fractions indicated that ABs, MVs, and Exo represented a relatively small part of FF lipids, since cell-free FF1 fraction and ffEV-free FF4 fraction showed limited differences. Therefore, free FA and lipoproteins likely represented the larger part of FF lipids. However, FF depleted from MVs and Exo may contain EVs smaller than exosome-like nanovesicles, which were not precipitated at the 100,000× *g* that was applied here. Such very small nanoparticles could be pelleted only at a higher speed (200,000× *g*), as was reported for fetal bovine serum and the secretions from human cell lines [53]. Nevertheless, high lipid variations that were observed here in ffEV fractions between the LF and SF suggested significant involvement of extracellular vesicles to the lipid composition of FF and discrimination of the follicles observed by MSI.

### 4.3. Lipids in MVs and Exo-Enriched Fractions Differed between the Large and Small Follicles

Morphological characterization of ffEV preparations demonstrated that MV-enriched fractions contained very heterogeneous vesicles bearing the remnants of different cell organelles. Exo fractions were more homogenous but could be contaminated with co-precipitated lipoproteins, notably HDLs, as was shown for human plasma EVs prepared by ultracentrifugation [54]. Interestingly, comparative lipidomics between human plasma and serum lipoproteins and EVs revealed that the EVs contained eight times fewer lipids per protein unit than the lipoproteins, and these groups had substantially different lipid profiles [55]. Indeed, blood EVs were enriched in LPC, LPE, and SM, which were over-abundant in the EVs compared to HDLs, whereas the relative abundance of PC and PE was higher in the HDLs [55]. In our study, Exo fractions were obtained by ultracentrifugation and thus were likely contaminated with the HDLs, in contrast to the MVs, precipitated by centrifugation at 12,000× *g*. Therefore, this difference contributed to MV and Exo lipid profiles and allowed discrimination of MVs and Exo fractions by PCA. Nevertheless, the clear discrimination of the LF and SF was observed regardless of the type of ffEVs. Overall, 66.7% of the features detected in ffEVs of SFs and LFs showed differential abundance between the MV SF, MV LF, Exo SF, and Exo LF groups (*p* < 0.01). In cows, the concentration of ffEVs decreased progressively as follicle size increased [15], whereas concentrations of apoptotic cells and their remnants, as well as HDL concentrations, were higher in the LFs compared to smaller ones [11]. Significant differences in FA composition of follicular fluids between the large dominant and small subordinate follicles in cows were reported earlier in different works [11,12,13,25]. The most exhaustive lipidomic study was performed on FF depleted from the cells and apoptotic remnants from the individual follicles of different sizes and revealed that 55% of FF lipids were differently abundant between LFs and SFs [14]. Among these differential lipids, mainly LPC and PC were more abundant in FF from the LFs, whereas diacylglycerols, SM, PE, and PI were overrepresented in the SFs. Therefore, FF differential lipids could originate either from the lipoproteins or from ffEVs.

Here, the determined differential lipids between SFs and LFs included the main lipid classes, but the most variations were observed for PC, SM, numerous lysophospholipids, CE, and ceramides. Follicular fluid EVs from the SF were particularly enriched in PC, SM, PE, PI, and their lysoforms. The ffEVs from the LF showed a higher abundance of PC, LPC, LPE, LPI, LPS, carnitines, and ceramides. Indeed, LPC, LPS, and LPI are involved in different signaling functions, stimulating cell proliferation, differentiation, survival, and inflammation in different cells [56,57]. Different pathways, including carbon and FA energy metabolism, including the pentose phosphate pathway and FA oxidation, steroidogenesis, PI-signaling, and n-3 and n-6 polyunsaturated FA metabolism, were enriched in the ffEV lipids identified here. Most of these pathways are known to be very important in female reproduction functions, and several of them were reported to be involved in folliculogenesis [1,3], ovarian energy metabolism [58,59,60], steroidogenesis by follicular theca and granulosa cells [16,61], oocyte growth and maturation [2,62,63,64], and preterm labor and delivery [65]. Moreover, in dairy cows, metabolic status was associated with ffEV-coupled miRNAs, which were involved in various pathways associated with follicular growth and oocyte maturation [66] and suggested the potential involvement of ffEVs in oocyte developmental competence [6]. Several LPIs present in Exo were also detected in the oocytes [16].

Follicular fluid exosomes are considered a specific tool of molecular exchange between the follicular cells and the oocyte [6,67], and their lipid cargo may be involved in the regulation of oocyte maturation events and influence oocyte quality [48]. Indeed, supplementation of bovine oocytes with ffEV preparations during in vitro maturation improved their competence in embryo development in vitro [68,69,70]. According to our pathway analysis, lipid species differentially abundant in ffEVs between the LFs and SFs were involved in different processes, and several of them are tightly related to pre-ovulation follicular development and oocyte maturation. Indeed, the metabolism of linoleic, alpha-linolenic, and arachidonic acids is crucial for oocyte competence and female fertility [71,72,73]. Glycerophospholipid, sphingolipid, and steroid metabolism were related to inflammation processes inside the preovulatory follicles [36,74], and the pathways, including phosphatidylinositol and inositol phosphate metabolism, corroborated the role of EVs as signaling mediators [6,75,76].

In summary, the contribution of extracellular vesicle lipids seems to be substantial to FF lipid composition. Substantial differences in FF lipid composition between the follicles of different sizes include the lipids from both vesicular and non-vesicular fractions and could be revealed by MSI at the ovarian sections, thus discriminating the dominant follicle from subordinate smaller follicles.

## Figures and Tables

**Table 1:** Putative annotations of lipids differentially abundant between cellular and extracellular fractions of follicular fluid.

Cluster	*m*/*z*	*p*-Value	Lipid Ion (Carbons: Unsaturation)
1	*m*/*z* 794.58+	7.94 × 10^−4^	PC(37:5)/PC(O-38:5)/PC(P-38:4)/PE(40:5)
1	*m*/*z* 808.58+	9.693 × 10^−3^	PC(38:5)/PE(41:5)/PE(P-42:4)
1	*m*/*z* 854.56+	3.56 × 10^−2^	PC(42:10)/PC(41:3)/PC(O-42:3)/PC(P-42:2)/PE(44:3)
1	*m*/*z* 882.59+	4.19 × 10^−2^	PC(44:10)/PC(O-44:3)
1	*m*/*z* 613.19−	3.10 × 10^−2^	LPI(19:0)/LPI(O-20:0)
2	*m*/*z* 675.56+	4.33 × 10^−2^	SM(d32:1)
2	*m*/*z* 689.57+	3.11 × 10^−2^	SM(d33:1)
2	*m*/*z* 806.57+	4.63 × 10^−2^	PC(38:6)/PE(41:6)/PE(O-42:6)
2	*m*/*z* 830.56+	3.27 × 10^−2^	PC(40:8)/PC(39:1)/PC(O-40:1)/PC(P-40:0)/PE(42:1)
2	*m*/*z* 836.62+	2.59 × 10^−2^	PC(40:5)/PE(44:12)
3	*m*/*z* 672.43+	6.06 × 10^−3^	PE(31:3)
3	*m*/*z* 726.56+	4.05 × 10^−3^	PC(32:4)/-/PE(35:4)/PE(O-36:4)/PE(P-36:3)
3	*m*/*z* 727.56+	1.22 × 10^−2^	SM(d36:3)
3	*m*/*z* 728.53+	1.38 × 10^−2^	PC(32:3)/PC(P-33:2)/PE(35:3)/PE(O-36:3)/PE(P-36:2)
3	*m*/*z* 326.18−	1.50 × 10^−2^	LPC(4:0)/PC(O-4:0)/LPC(O-5:0)
4	*m*/*z* 340.31−	1.98 × 10^−2^	LPC(5:0)
5	*m*/*z* 620.21+	2.04 × 10^−2^	PE(27:1)/PE(P-28:0)
5	*m*/*z* 617.18−	6.59 × 10^−3^	LPI(20:5)
5	*m*/*z* 618.19−	1.66 × 10^−2^	PE(27:1)/PE(P-28:0)
5	*m*/*z* 631.17−	8.66 × 10^−3^	None
5	*m*/*z* 650.14−	3.91 × 10^−2^	PS(26:0)/LPS(-OMe-226:1)
5	*m*/*z* 917.26−	4.82 × 10^−2^	PI(40:2)
6	*m*/*z* 730.54+	3.89 × 10^−2^	PC(32:2)/PC(O-33:2)/PC(P-33:1)/PE(35:2)/PE(P-36:1)
6	*m*/*z* 733.57+	4.14 × 10^−2^	SM(d36:0)
6	*m*/*z* 790.63+	1.61 × 10^−3^	PC(37:7)/PC(P-38:6)/PC(36:0)/PE(39:0)/PE(40:7)

**Table 2:** Ranked pathways of the lipids detected in ffEVs.

Pathways (*m*/*z*- Features)	Total	Enrichment	*p-Value (Fisher)*
Glycosylphosphatidylinositol (GPI)-anchor biosynthesis	6	83.4	0.040
Glycerophospholipid metabolism	156	6.4	0.012
Cytochrome P450 metabolism	53	9.4	0.29
Vitamin B9 (folate) metabolism	33	15.2	0.17
Vitamin A (retinol) metabolism	67	7.5	0.28
Phosphatidylinositol phosphate metabolism	59	8.5	0.12
Carbon fixation	10	8.3	0.65
Galactose metabolism	41	4.1	0.16
Putative anti-inflammatory metabolites formation from EPA (C20:5)	27	3.1	0.96
Fatty acid oxidation (peroxisome)	28	3.0	0.40
Fructose and mannose metabolism	33	2.5	0.88
Fatty acid oxidation	35	2.4	0.40
Pentose phosphate pathway	37	2.3	0.40
Omega-3 fatty acid metabolism	39	2.1	0.65
Glycolysis and Gluconeogenesis	49	1.7	0.99
Sialic acid metabolism	107	1.6	0.53
Squalene and cholesterol biosynthesis	55	1.5	0.79
Omega-6 fatty acid metabolism	55	1.5	0.40
Phosphatidylinositol phosphate metabolism	59	1.4	0.96
Carnitine shuttle	72	1.2	0.65
Fatty acid activation	74	1.1	0.40
Prostaglandin formation from arachidonate	78	1.1	0.40

**Table 3:** Differential lipid features detected between MVs and Exo fractions (*p* < 0.05, fold change > 2).

Lipids More Abundant in Exo	*p*-Value	Ratio Exo/MVs	Lipid Ion (Carbons: Unsaturation)
*m*/*z* 136.13+	4.60 × 10^−2^	2.46	-
*m*/*z* 144.13−	1.47 × 10^−2^	4.83	-
*m*/*z* 188.13−	5.68 × 10^−3^	3.59	-
*m*/*z* 246.11−	9.00 × 10^−3^	6.39	-
*m*/*z* 362.15−	1.34 × 10^−2^	2.99	LPC(4:0)/PC(O-4:0)
*m*/*z* 616.48−	9.49 × 10^−3^	2.47	LPS(22:0)
*m*/*z* 675.55+	1.85 × 10^−2^	2.00	SM(d32:1)/CE(18:0)
*m*/*z* 687.54−	6.10 × 10^−3^	2.32	SM(d33:1)/LPI(22:2)
*m*/*z* 688.54−	6.77 × 10^−3^	2.28	PE(32:1)
*m*/*z* 689.54−	7.42 × 10^−3^	2.22	SM(d33:0)/LPI(22:1)
*m*/*z* 689.56+	1.12 × 10^−2^	2.03	SM(d33:1)/CE(19:0)
*m*/*z* 703.58+	1.40 × 10^−2^	2.13	SM(d34:1)/CE(20:0)
*m*/*z* 704.58+	1.36 × 10^−2^	2.13	PC or PE
*m*/*z* 705.59+	1.54 × 10^−2^	2.13	PC or PE
*m*/*z* 715.55−	4.55 × 10^−3^	2.18	SM(d35:1)
*m*/*z* 731.60+	9.35 × 10^−3^	2.23	SM(d36:1)
*m*/*z* 797.64−	1.70 × 10^−3^	2.19	SM(d41:2)
*m*/*z* 798.64−	2.51 × 10^−3^	2.07	PE(40:2)
*m*/*z* 815.54−	1.21 × 10^−2^	2.38	SM(d42:0) or PI
*m*/*z* 816.54−	1.42 × 10^−2^	2.06	Su (d18:2/C19:1)
*m*/*z* 853.59−	2.91 × 10^−4^	2.14	PI
*m*/*z* 854.59−	3.96 × 10^−4^	2.06	PC, PE, or PS
*m*/*z* 925.65−	8.91 × 10^−3^	2.09	PI
*m*/*z* 963.72−	5.21 × 10^−4^	2.08	PI
*m*/*z* 104.18+	3.75 × 10^−5^	0.43	Choline
*m*/*z* 190.86+	5.72 × 10^−3^	0.40	-
*m*/*z* 212.08+	4.21 × 10^−5^	0.43	-
*m*/*z* 264.17+	2.31 × 10^−11^	0.16	-
*m*/*z* 277.02−	1.68 × 10^−2^	0.37	-
*m*/*z* 280.18+	7.25 × 10^−4^	0.21	-
*m*/*z* 325.27−	3.65 × 10^−2^	0.48	-
*m*/*z* 381.13+	5.57 × 10^−4^	0.31	-
*m*/*z* 393.07−	2.86 × 10^−2^	0.28	-
*m*/*z* 509.10−	1.859 × 10^−2^	0.27	-
*m*/*z* 599.34−	3.87 × 10^−2^	0.39	LPI(18:0)
*m*/*z* 660.09−	2.76 × 10^−2^	0.30	LPS(24:0)
*m*/*z* 672.42+	4.43 × 10^−3^	0.45	PE/PC/Cer(d18:1/24:0)

**Table 4:** Representative lipid features in ffEVs differentially abundant between large and small follicles (*p* < 0.05 and fold change > 2).

Lipids More Abundant in LF	*p*-Value	Ratio LF/SF	Lipid Ion
			(Carbons: Unsaturation)
*m*/*z* 312.27−	6.46 × 10^−4^	2.91	LPC(3:0)/PC(O-3:0)
*m*/*z* 327.44+	2.55 × 10^−3^	3.87	-
*m*/*z* 340.30−	2.33 × 10^−3^	3.35	LPC(5:0)
*m*/*z* 358.07+	2.23 × 10^−3^	9.88	-
*m*/*z* 393.07−	5.03 × 10^−3^	4.44	-
*m*/*z* 484.24−	1.50 × 10^−3^	2.5	LPS(14:0)CAR(20:3)
*m*/*z* 498.24−	1.29 × 10^−3^	2.78	LPE(20:5)//LPE(17:2)
*m*/*z* 509.10−	2.51 × 10^−3^	4.7	-
*m*/*z* 660.09−	2.91 × 10^−4^	6.16	LPS(22:0)
*m*/*z* 672.42+	2.15 × 10^−3^	2.25	Cer(d18:1/24:0)
*m*/*z* 526.33−	1.87 × 10^−6^	0.18	LPE(22:5)
*m*/*z* 528.33−	1.80 × 10^−7^	0.28	LPE(22:4)
*m*/*z* 597.32−	5.20 × 10^−3^	0.22	PS(41a:7)
*m*/*z* 599.34−	7.05 × 10^−4^	0.16	LPI(18:0)
*m*/*z* 675.55+	1.30 × 10^−3^	0.36	CE(17:1)
*m*/*z* 701.55+	2.12 × 10^−3^	0.45	CE(20:1)
*m*/*z* 706.54+	1.69 × 10^−5^	0.3	PE(32:4)
*m*/*z* 722.50−	5.06 × 10^−6^	0.26	PE 34a:0
*m*/*z* 731.60+	5.15 × 10^−4^	0.31	SM(d36:1)
*m*/*z* 734.57+	1.11 × 10^−5^	0.25	PC(32:0)
*m*/*z* 748.52−	1.15 × 10^−5^	0.27	PE(P-38:5)/PE(O-38:6)
*m*/*z* 749.51−	4.04 × 10^−6^	0.27	PI(28:2)
*m*/*z* 750.53−	1.30 × 10^−5^	0.28	PE(P-38:4)
*m*/*z* 757.55+	3.66 × 10^−13^	0.29	SM(d38:2)
*m*/*z* 760.50−	2.90 × 10^−5^	0.27	PE(38:7)
*m*/*z* 768.53−	1.20 × 10^−7^	0.29	PE(38a:4)
*m*/*z* 788.53−	2.02 × 10^−5^	0.3	PE(40:7)
*m*/*z* 790.53−	1.91 × 10^−6^	0.32	PE(40:6)
*m*/*z* 794.54−	9.43 × 10^−8^	0.31	PS(P-38:4)
*m*/*z* 813.68+	8.16 × 10^−6^	0.25	SM(d42:2)
*m*/*z* 815.69+	1.19 × 10^−6^	0.26	SM(d42:1)

**Table 5:** Pathway enriched in differentially abundant lipids between the LFs and SFs.

Pathways	Total	Hits	Enrichment	Raw *p*-Value	Impact
Glycerophospholipid metabolism	36	3	25.8	1.12 × 10^−4^	0.218
Glycosylphosphatidylinositol (GPI)-anchor biosynthesis	14	2	44.3	7.46 × 10^−4^	0.004
Sphingolipid metabolism	21	2	29.5	1.71 × 10^−3^	0.269
Linoleic acid metabolism	5	1	62.0	1.60 × 10^−2^	0
Alpha-linolenic acid metabolism	13	1	23.8	4.13 × 10^−2^	0
Phosphatidylinositol signaling system	28	1	11.1	8.72 × 10^−2^	0.097
Inositol phosphate metabolism	30	1	10.3	9.32 × 10^−2^	0.078

## Data Availability

Supplementary data are available on the MDPI site.

## Supplementary Materials

The following supporting information can be downloaded at https://www.mdpi.com/article/10.3390/metabo13091001/s1, Table S1: Putative annotations of lipid features detected by MS imaging on bovine ovarian section using HABA matrix; Table S2: Putative annotations of lipid features detected in follicular fluid FF1, FF2, FF3 and F4 fractions; Table S3: Abundance of dfferential lipid species in FF1, FF2, FF3 and FF4 samples; Table S4: Putative annotations of lipid features detected in follicular fluid "solid" fractions (cells and ffEVs); Table S5: Abundance of dfferential lipid species in cellular and ffEV enriched fractions; Table S6: Putative annotations of lipid features detected in MV and Exo; Table S7: Differentially abundant lipids in ffEVs from large and small follicles in cows; Table S8: Differentially abundant lipids in MV and Exo from large and small follicles in cows; Table S9: Putative annotations of lipid features in MV and Exo differentially abundant between large and small follicles.

## References

[1] Dalbies-Tran R., Cadoret V., Desmarchais A., Elis S., Maillard V., Monget P., Monniaux D., Reynaud K., Saint-Dizier M., Uzbekova S. (2020). A comparative analysis of oocyte development in mammals. Cells.

[2] Dunning K.R., Russell D.L., Robker R.L. (2014). Lipids and oocyte developmental competence: The role of fatty acids and b-oxidation. Reproduction.

[3] Collado-Fernandez E., Picton H.M., Dumollard R. (2012). Metabolism throughout follicle and oocyte development in mammals. Int. J. Dev. Biol..

[4] Yanez-Mo M., Siljander P.R., Andreu Z., Zavec A.B., Borras F.E., Buzas E.I., Buzas K., Casal E., Cappello F., Carvalho J. (2015). Biological properties of extracellular vesicles and their physiological functions. J. Extracell. Vesicles.

[5] Saeed-Zidane M., Linden L., Salilew-Wondim D., Held E., Neuhoff C., Tholen E., Hoelker M., Schellander K., Tesfaye D. (2017). Cellular and exosome mediated molecular defense mechanism in bovine granulosa cells exposed to oxidative stress. PLoS ONE.

[6] Tesfaye D., Hailay T., Salilew-Wondim D., Hoelker M., Bitseha S., Gebremedhn S. (2020). Extracellular vesicle mediated molecular signaling in ovarian follicle: Implication for oocyte developmental competence. Theriogenology.

[7] Skotland T., Sagini K., Sandvig K., Llorente A. (2020). An emerging focus on lipids in extracellular vesicles. Adv. Drug Deliv. Rev..

[8] Lonergan P., Monaghan P., Rizos D., Boland M.P., Gordon I. (1994). Effect of follicle size on bovine oocyte quality and developmental competence following maturation, fertilization, and culture in vitro. Mol. Reprod. Dev..

[9] Blondin P., Sirard M.A. (1995). Oocyte and follicular morphology as determining characteristics for developmental competence in bovine oocytes. Mol. Reprod. Dev..

[10] Monniaux D., Cadoret V., Clément F., Dalbies Tran R., Elis S., Fabre S., Maillard V., Monget P., Uzbekova S. (2018). Folliculogenesis. Reference Module in Biomedical Sciences.

[11] Brantmeier S.A., Grummer R.R., Ax R.L. (1987). Concentrations of high density lipoproteins vary among follicular sizes in the bovine. J. Dairy Sci..

[12] Leroy J.L., Vanholder T., Delanghe J.R., Opsomer G., Van Soom A., Bols P.E., de Kruif A. (2004). Metabolite and ionic composition of follicular fluid from different-sized follicles and their relationship to serum concentrations in dairy cows. Anim. Reprod. Sci..

[13] Renaville B., Bacciu N., Comin A., Motta M., Poli I., Vanini G., Prandi A. (2010). Plasma and follicular fluid fatty acid profiles in dairy cows. Reprod. Domest. Anim..

[14] Bertevello P.S., Teixeira-Gomes A.P., Labas V., Cordeiro L., Blache M.C., Papillier P., Singina G., Uzbekov R., Maillard V., Uzbekova S. (2020). Maldi-tof mass spectrometry revealed significant lipid variations in follicular fluid and somatic follicular cells but not in enclosed oocytes between the large dominant and small subordinate follicles in bovine ovary. Int. J. Mol. Sci..

[15] Navakanitworakul R., Hung W.T., Gunewardena S., Davis J.S., Chotigeat W., Christenson L.K. (2016). Characterization and small rna content of extracellular vesicles in follicular fluid of developing bovine antral follicles. Sci. Rep..

[16] Bertevello P.S., Teixeira-Gomes A.P., Seyer A., Vitorino Carvalho A., Labas V., Blache M.C., Banliat C., Cordeiro L.A.V., Duranthon V., Papillier P. (2018). Lipid identification and transcriptional analysis of controlling enzymes in bovine ovarian follicle. Int J Mol Sci.

[17] Baker T.C., Han J., Borchers C.H. (2017). Recent advancements in matrix-assisted laser desorption/ionization mass spectrometry imaging. Curr. Opin. Biotechnol..

[18] Perry W.J., Patterson N.H., Prentice B.M., Neumann E.K., Caprioli R.M., Spraggins J.M. (2020). Uncovering matrix effects on lipid analyses in maldi imaging mass spectrometry experiments. J. Mass Spectrom..

[19] Campbell D.I., Ferreira C.R., Eberlin L.S., Cooks R.G. (2012). Improved spatial resolution in the imaging of biological tissue using desorption electrospray ionization. Anal. Bioanal. Chem..

[20] Uzbekova S., Elis S., Teixeira-Gomes A.P., Desmarchais A., Maillard V., Labas V. (2015). Maldi mass spectrometry imaging of lipids and gene expression reveals differences in fatty acid metabolism between follicular compartments in porcine ovaries. Biology.

[21] Cordeiro F.B., Jarmusch A.K., Leon M., Ferreira C.R., Pirro V., Eberlin L.S., Hallett J., Miglino M.A., Cooks R.G. (2020). Mammalian ovarian lipid distributions by desorption electrospray ionization-mass spectrometry (desi-ms) imaging. Anal. Bioanal. Chem..

[22] Leroy J.L., Vanholder T., Mateusen B., Christophe A., Opsomer G., de Kruif A., Genicot G., Van Soom A. (2005). Non-esterified fatty acids in follicular fluid of dairy cows and their effect on developmental capacity of bovine oocytes in vitro. Reproduction.

[23] McNatty K.P., Heath D.A., Henderson K.M., Lun S., Hurst P.R., Ellis L.M., Montgomery G.W., Morrison L., Thurley D.C. (1984). Some aspects of thecal and granulosa cell function during follicular development in the bovine ovary. J. Reprod. Fertil..

[24] Monniaux D., Huet C., Besnard N., Clement F., Bosc M., Pisselet C., Monget P., Mariana J.C. (1997). Follicular growth and ovarian dynamics in mammals. J. Reprod. Fertil. Suppl..

[25] Argov N., Sklan D. (2004). Expression of mrna of lipoprotein receptor related protein 8, low density lipoprotein receptor, and very low density lipoprotein receptor in bovine ovarian cells during follicular development and corpus luteum formation and regression. Mol. Reprod. Dev..

[26] Uzbekova S., Bertevello P.S., Dalbies-Tran R., Elis S., Labas V., Monget P., Teixeira-Gomes A.-P. (2022). Metabolic exchanges between the oocyte and its environment: Focus on lipids. Reprod. Fertil. Dev..

[27] Guo X., Wang X., Di R., Liu Q., Hu W., He X., Yu J., Zhang X., Zhang J., Broniowska K. (2018). Metabolic effects of fecb gene on follicular fluid and ovarian vein serum in sheep (ovis aries). Int. J. Mol. Sci..

[28] Conti M., Hsieh M., Zamah A.M., Oh J.S. (2012). Novel signaling mechanisms in the ovary during oocyte maturation and ovulation. Mol. Cell. Endocrinol..

[29] Vigneron C., Perreau C., Dupont J., Uzbekova S., Prigent C., Mermillod P. (2004). Several signaling pathways are involved in the control of cattle oocyte maturation. Mol. Reprod. Dev..

[30] Schiller J., Süß R., Arnhold J., Fuchs B., Leßig J., Müller M., Petković M., Spalteholz H., Zschörnig O., Arnold K. (2004). Matrix-assisted laser desorption and ionization time-of-flight (maldi-tof) mass spectrometry in lipid and phospholipid research. Prog. Lipid Res..

[31] Fuchs B., Schiller J. (2008). Maldi-tof ms analysis of lipids from cells, tissues and body fluids. Lipids Health Dis..

[32] Lagarrigue M., Lavigne R., Chaurand P., Com E., Pineau C., Guével B. (2011). Matrix-assisted laser desorption/ionization imaging mass spectrometry: A promising technique for reproductive research1. Biol. Reprod..

[33] Soler L., Uzbekova S., Blesbois E., Druart X., Labas V. (2020). Intact cell maldi-tof mass spectrometry, a promising proteomic profiling method in farm animal clinical and reproduction research. Theriogenology.

[34] Thompson J.E. (2022). Matrix-assisted laser desorption ionization-time-of-flight mass spectrometry in veterinary medicine: Recent advances (2019–present). Vet. World.

[35] Ferreira C.R., Saraiva S.A., Catharino R.R., Garcia J.S., Gozzo F.C., Sanvido G.B., Santos L.F.A., Lo Turco E.G., Pontes J.H.F., Basso A.C. (2010). Single embryo and oocyte lipid fingerprinting by mass spectrometry. J. Lipid Res..

[36] Santos P.H., Fontes P.K., Franchi F.F., Nogueira M.F., Belaz K.R., Tata A., Eberlin M.N., Sudano M.J., Barros C.M., Castilho A.C. (2017). Lipid profiles of follicular fluid from cows submitted to ovarian superstimulation. Theriogenology.

[37] Cordeiro F.B., Montani D.A., Pilau E.J., Gozzo F.C., Fraietta R., Turco E.G.L. (2018). Ovarian environment aging: Follicular fluid lipidomic and related metabolic pathways. J. Assist. Reprod. Genet..

[38] Vireque A.A., Tata A., Roberta K., Belaz A., Gabriel J., Grázia V., Santos F.N., Arnold D.R., Basso A.C., Eberlin M.N. (2017). Maldi mass spectrometry reveals that cumulus cells modulate the lipid profile of in vitro- matured bovine oocytes. Syst. Biol. Reprod. Med..

[39] Gonçalves R.F., Ferreira M.S., Oliveira D.N.d., Canevarolo R., Achilles M.A., D’Ercole D.L., Bols P.E., Visintin J.A., Killian G.J., Catharino R.R. (2014). Analysis and characterisation of bovine oocyte and embryo biomarkers by matrix-assisted desorption ionisation mass spectrometry imaging. Reprod. Fertil. Dev..

[40] Freret S., Oseikria M., Le Bourhis D., Desmarchais A., Briant E., Desnoes O., Dupont M., Le Berre L., Ghazouani O., Bertevello P.S. (2019). Effects of a n-3 pufa enriched diet on embryo production in dairy cows. Reproduction.

[41] Elis S., Oseikria M., Vitorino Carvalho A., Bertevello P.S., Corbin E., Teixeira-Gomes A.P., Lecardonnel J., Archilla C., Duranthon V., Labas V. (2017). Docosahexaenoic acid mechanisms of action on the bovine oocyte-cumulus complex. J. Ovarian Res..

[42] Pfrieger F.W., Vitale N. (2018). Cholesterol and the journey of extracellular vesicles. J. Lipid Res..

[43] Elsherbini A., Bieberich E., Chalfant C.E., Fisher P.B. (2018). Chapter five—Ceramide and exosomes: A novel target in cancer biology and therapy. Advances in Cancer Research.

[44] Jaspard B., Barbaras R., Manent J., Parinaud J., Chap H., Perret B. (1996). Biochemical characterization of pre-β 1 high-density lipoprotein from human ovarian follicular fluid: Evidence for the presence of a lipid core. Biochemistry.

[45] Arias A., Quiroz A., Santander N., Morselli E., Busso D. (2022). Implications of high-density cholesterol metabolism for oocyte biology and female fertility. Front. Cell Dev. Biol..

[46] Volpe A., Coukos G., Uccelli E., Droghini F., Adamo R., Artini P.G. (1991). Follicular fluid lipoproteins in preovulatory period and their relationship with follicular maturation and progesterone production by human granulosa-luteal cells in vivo and in vitro. J. Endocrinol. Investig..

[47] Huang Q., Liu Y., Yang Z., Xie Y., Mo Z. (2019). The effects of cholesterol metabolism on follicular development and ovarian function. Curr. Mol. Med..

[48] da Silveira J.C., Andrade G.M., Simas R.C., Martins-Junior H.A., Eberlin M.N., Smith L.C., Perecin F., Meirelles F.V. (2021). Lipid profile of extracellular vesicles and their relationship with bovine oocyte developmental competence: New players in intra follicular cell communication. Theriogenology.

[49] Haraszti R.A., Didiot M.C., Sapp E., Leszyk J., Shaffer S.A., Rockwell H.E., Gao F., Narain N.R., DiFiglia M., Kiebish M.A. (2016). High-resolution proteomic and lipidomic analysis of exosomes and microvesicles from different cell sources. J. Extracell. Vesicles.

[50] Paolino G., Buratta S., Mercuri S.R., Pellegrino R.M., Urbanelli L., Emiliani C., Bertuccini L., Iosi F., Huber V., Brianti P. (2022). Lipidic profile changes in exosomes and microvesicles derived from plasma of monoclonal antibody-treated psoriatic patients. Front. Cell Dev. Biol..

[51] Record M., Silvente-Poirot S., Poirot M., Wakelam M.J.O. (2018). Extracellular vesicles: Lipids as key components of their biogenesis and functions. J. Lipid Res..

[52] Skotland T., Sandvig K., Llorente A. (2017). Lipids in exosomes: Current knowledge and the way forward. Prog. Lipid Res..

[53] Lee S.S., Won J.H., Lim G.J., Han J., Lee J.Y., Cho K.O., Bae Y.K. (2019). A novel population of extracellular vesicles smaller than exosomes promotes cell proliferation. Cell Commun. Signal. CCS.

[54] Yuana Y., Levels J., Grootemaat A., Sturk A., Nieuwland R. (2014). Co-isolation of extracellular vesicles and high-density lipoproteins using density gradient ultracentrifugation. J. Extracell. Vesicles.

[55] Sun Y., Saito K., Saito Y. (2019). Lipid profile characterization and lipoprotein comparison of extracellular vesicles from human plasma and serum. Metabolites.

[56] Law S.H., Chan M.L., Marathe G.K., Parveen F., Chen C.H., Ke L.Y. (2019). An updated review of lysophosphatidylcholine metabolism in human diseases. Int. J. Mol. Sci..

[57] Drzazga A., Sowinska A., Koziolkiewicz M. (2014). Lysophosphatidylcholine and lysophosphatidylinosiol--novel promissing signaling molecules and their possible therapeutic activity. Acta Pol. Pharm..

[58] Dupont J., Reverchon M., Cloix L., Froment P., Rame C. (2012). Involvement of adipokines, ampk, pi3k and the ppar signaling pathways in ovarian follicle development and cancer. Int. J. Dev. Biol..

[59] Moallem U. (2018). Invited review: Roles of dietary n-3 fatty acids in performance, milk fat composition, and reproductive and immune systems in dairy cattle. J. Dairy Sci..

[60] Elis S., Freret S., Desmarchais A., Maillard V., Cognie J., Briant E., Touze J.L., Dupont M., Faverdin P., Chajes V. (2016). Effect of a long chain n-3 pufa-enriched diet on production and reproduction variables in holstein dairy cows. Anim. Reprod. Sci..

[61] Hughes J., Kwong W.Y., Li D., Salter A.M., Lea R.G., Sinclair K.D. (2011). Effects of omega-3 and -6 polyunsaturated fatty acids on ovine follicular cell steroidogenesis, embryo development and molecular markers of fatty acid metabolism. Reproduction.

[62] Sanchez-Lazo L., Brisard D., Elis S., Maillard V., Uzbekov R., Labas V., Desmarchais A., Papillier P., Monget P., Uzbekova S. (2014). Fatty acid synthesis and oxidation in cumulus cells support oocyte maturation in bovine. Mol. Endocrinol..

[63] Nuttinck F., Gall L., Ruffini S., Laffont L., Clement L., Reinaud P., Adenot P., Grimard B., Charpigny G., Marquant-Le Guienne B. (2011). Ptgs2-related pge2 affects oocyte mapk phosphorylation and meiosis progression in cattle: Late effects on early embryonic development. Biol. Reprod..

[64] Marei W.F., Wathes D.C., Fouladi-Nashta A.A. (2009). The effect of linolenic acid on bovine oocyte maturation and development. Biol. Reprod..

[65] Peiris H.N., Vaswani K., Almughlliq F., Koh Y.Q., Mitchell M.D. (2017). Review: Eicosanoids in preterm labor and delivery: Potential roles of exosomes in eicosanoid functions. Placenta.

[66] Hailay T., Hoelker M., Poirier M., Gebremedhn S., Rings F., Saeed-Zidane M., Salilew-Wondim D., Dauben C., Tholen E., Neuhoff C. (2019). Extracellular vesicle-coupled mirna profiles in follicular fluid of cows with divergent post-calving metabolic status. Sci. Rep..

[67] Di Pietro C. (2016). Exosome-mediated communication in the ovarian follicle. J. Assist. Reprod. Genet..

[68] da Silveira J.C., Andrade G.M., Del Collado M., Sampaio R.V., Sangalli J.R., Silva L.A., Pinaffi F.V.L., Jardim I.B., Cesar M.C., Nogueira M.F.G. (2017). Supplementation with small-extracellular vesicles from ovarian follicular fluid during in vitro production modulates bovine embryo development. PLoS ONE.

[69] Machtinger R., Laurent L.C., Baccarelli A.A. (2016). Extracellular vesicles: Roles in gamete maturation, fertilization and embryo implantation. Hum. Reprod. Update.

[70] Singina G., Shedova E., Uzbekov R., Uzbekova S. (2022). Effect of different concentrations of follicular fluid exosome-like extracellular vesicles on in vitro oocyte maturation and embryo development in cattle. Annual Conference of the International Embryo Transfer Society IETS.

[71] Shaaker M., Rahimipour A., Nouri M., Khanaki K., Darabi M., Farzadi L., Shahnazi V., Mehdizadeh A. (2012). Fatty acid composition of human follicular fluid phospholipids and fertilization rate in assisted reproductive techniques. Iran. Biomed. J..

[72] González-Serrano A.F., Ferreira C.R., Pirro V., Lucas-Hahn A., Heinzmann J., Hadeler K.-G., Baulain U., Aldag P., Meyer U., Piechotta M. (2015). Effects of long-term dietary supplementation with conjugated linoleic acid on bovine oocyte lipid profile. Reprod. Fertil. Dev..

[73] Marei W.F., Wathes D.C., Fouladi-Nashta A.A. (2010). Impact of linoleic acid on bovine oocyte maturation and embryo development. Reproduction.

[74] Robker R.L., Wu L.L.Y., Yang X. (2011). Inflammatory pathways linking obesity and ovarian dysfunction. J. Reprod. Immunol..

[75] Sohel M.M.H., Hoelker M., Noferesti S.S., Salilew-Wondim D., Tholen E., Looft C., Rings F., Uddin M.J., Spencer T.E., Schellander K. (2013). Exosomal and non-exosomal transport of extra-cellular micrornas in follicular fluid: Implications for bovine oocyte developmental competence. PLoS ONE.

[76] da Silveira J.C., de Avila A., Garrett H.L., Bruemmer J.E., Winger Q.A., Bouma G.J. (2018). Cell-secreted vesicles containing micrornas as regulators of gamete maturation. J. Endocrinol..

